# Supplementary figures and images for: Fat body glycolysis defects inhibit mTOR and promote distant muscle disorganization through TNF-α/egr and ImpL2 signaling in Drosophila larvae
Source: EMBO Rep. 2024 Sep 9;25(10):4410–32. doi: 10.1038/s44319-024-00241-3 (PMC11467327; doi:10.1038/s44319-024-00241-3)

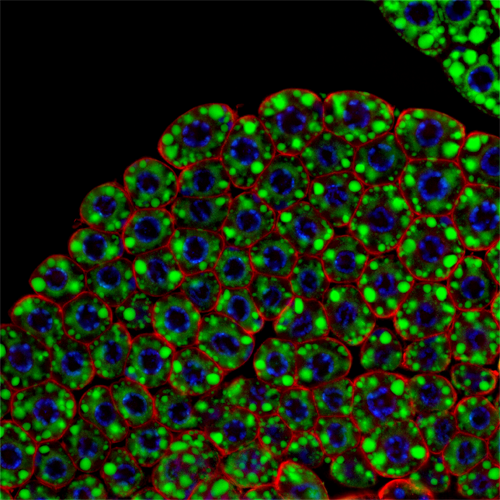

Supplement: Supplementary file 2 — Source data Fig. 1 [file 44319_2024_241_MOESM2_ESM.zip › Figure1/1B/lpp>eno-i.tif]

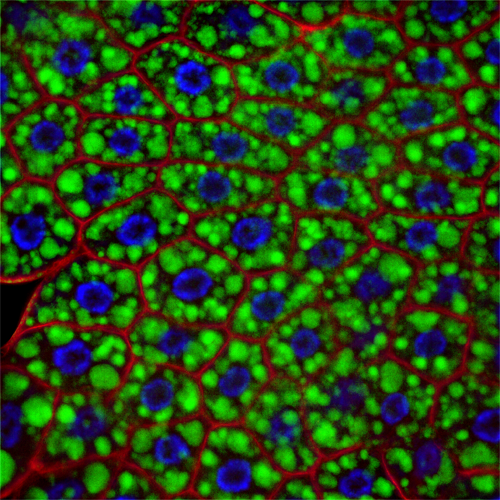

Supplement: Supplementary file 2 — Source data Fig. 1 [file 44319_2024_241_MOESM2_ESM.zip › Figure1/1B/lpp>pgk-i.tif]

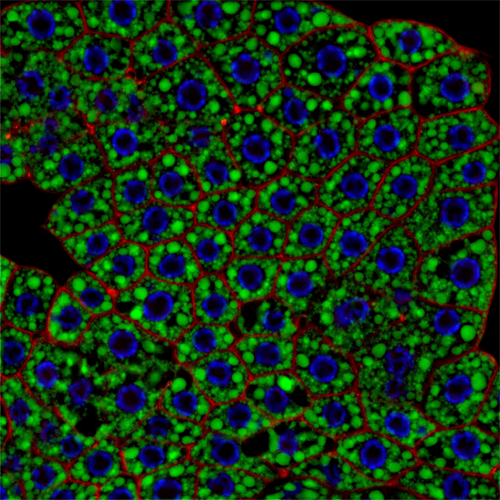

Supplement: Supplementary file 2 — Source data Fig. 1 [file 44319_2024_241_MOESM2_ESM.zip › Figure1/1B/lpp>pglym78-itif.tif]

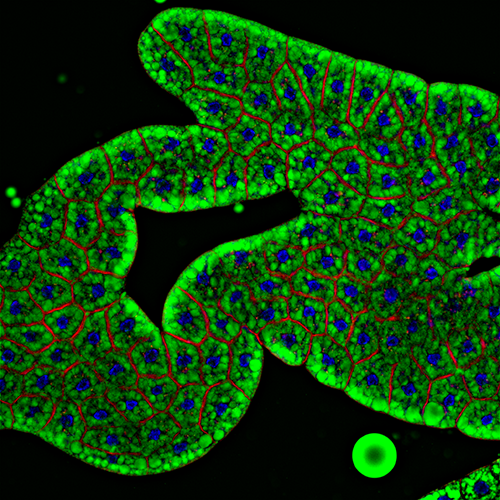

Supplement: Supplementary file 2 — Source data Fig. 1 [file 44319_2024_241_MOESM2_ESM.zip › Figure1/1B/lpp>pglym78-i_pyruvate food..tif]

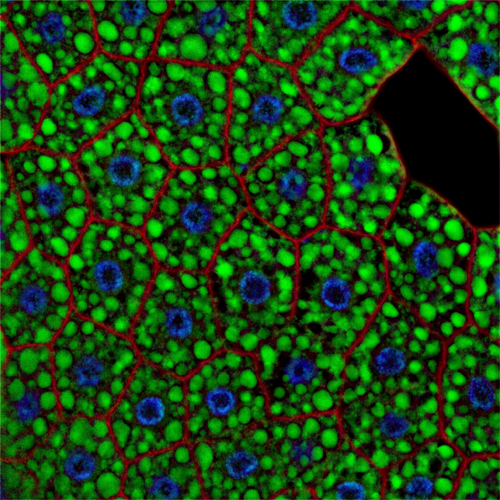

Supplement: Supplementary file 2 — Source data Fig. 1 [file 44319_2024_241_MOESM2_ESM.zip › Figure1/1B/lpp>w-i.tif]

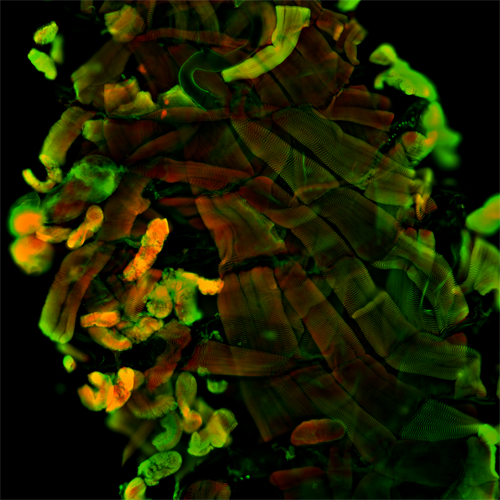

Supplement: Supplementary file 4 — Source data Fig. 3 [file 44319_2024_241_MOESM4_ESM.zip › Figure3/3A/lpp>eno-i.tif]

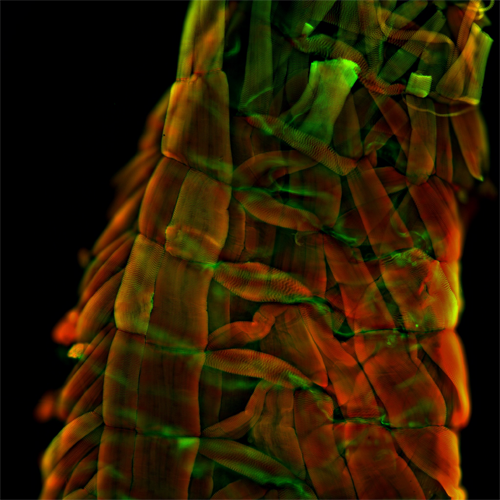

Supplement: Supplementary file 4 — Source data Fig. 3 [file 44319_2024_241_MOESM4_ESM.zip › Figure3/3A/lpp>pgk-i.tif]

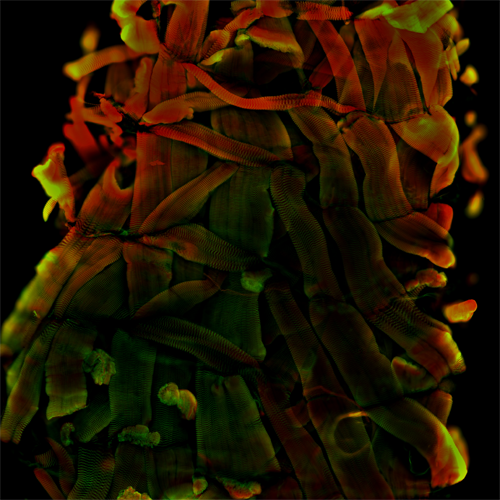

Supplement: Supplementary file 4 — Source data Fig. 3 [file 44319_2024_241_MOESM4_ESM.zip › Figure3/3A/Lpp>pglym78-i.tif]

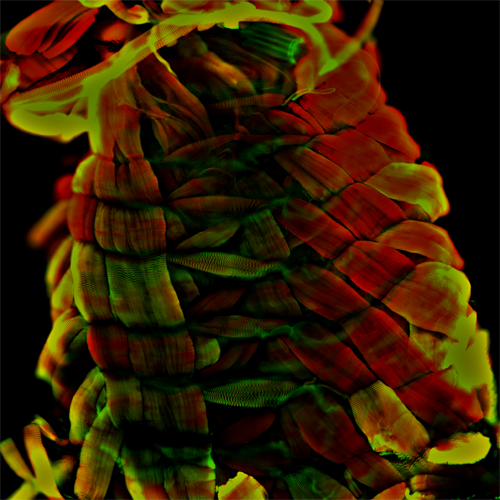

Supplement: Supplementary file 4 — Source data Fig. 3 [file 44319_2024_241_MOESM4_ESM.zip › Figure3/3A/lpp>w-i.tif]

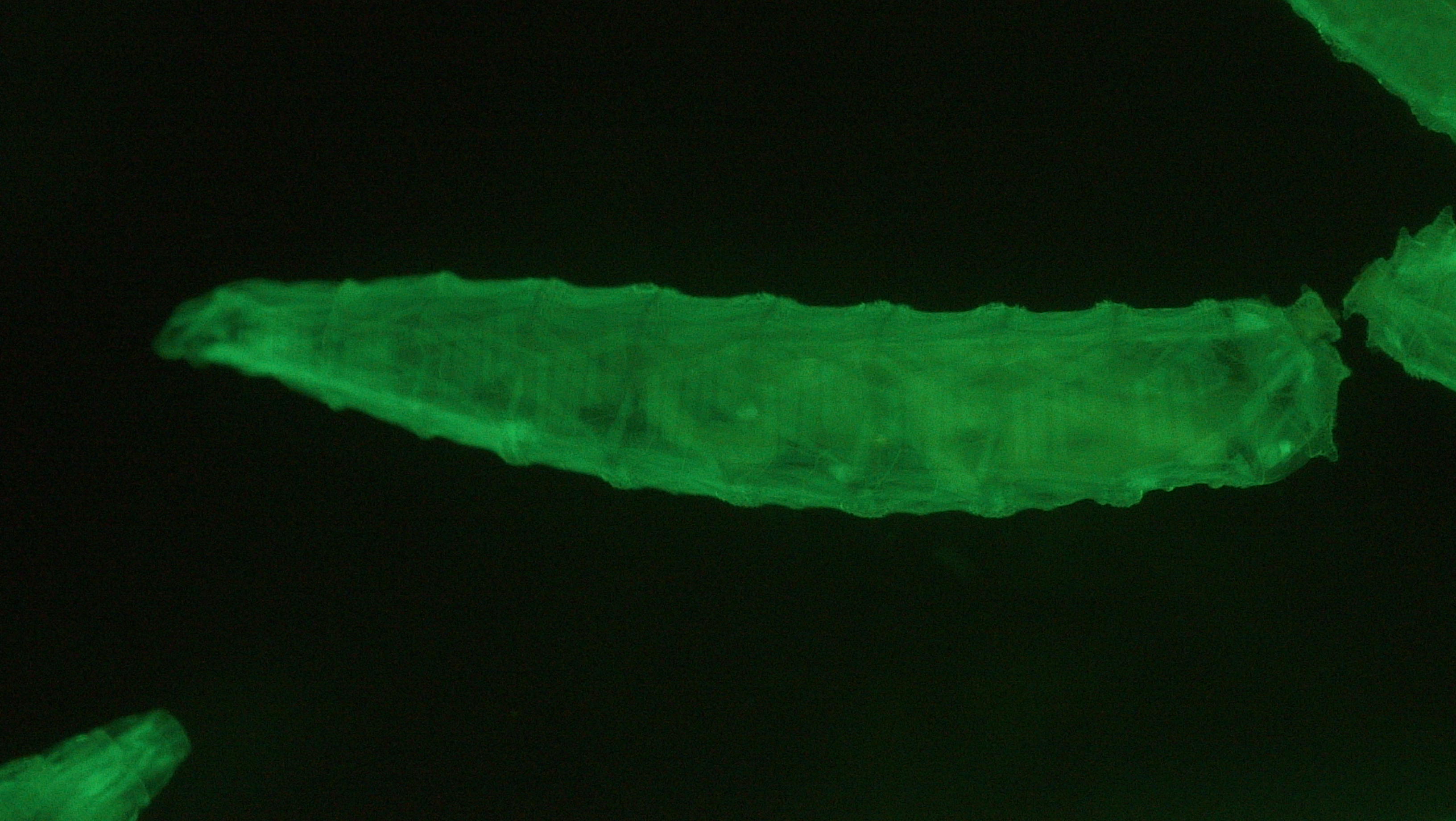

Supplement: Supplementary file 4 — Source data Fig. 3 [file 44319_2024_241_MOESM4_ESM.zip › Figure3/3B/lpp>eno-i.JPG]

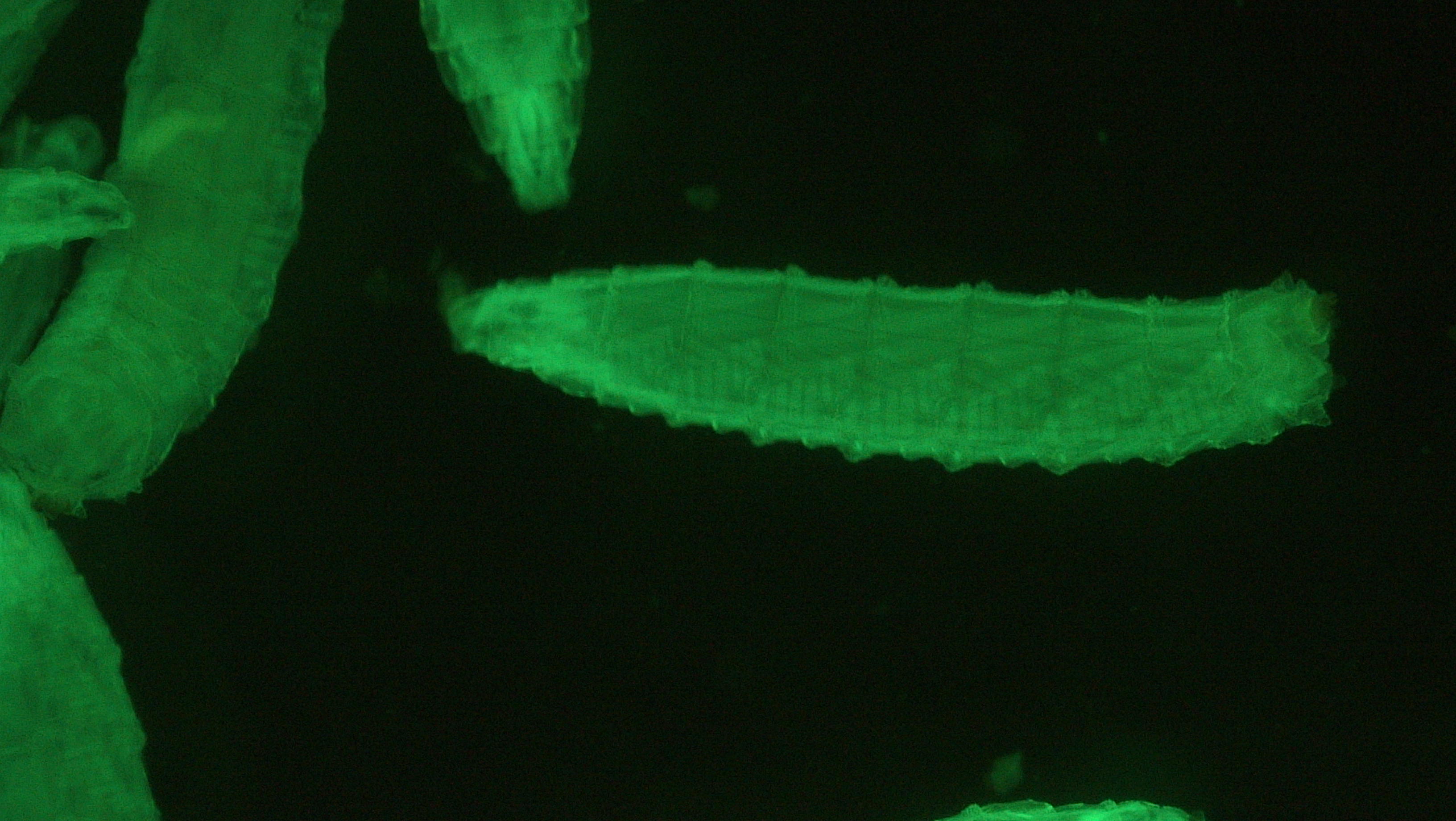

Supplement: Supplementary file 4 — Source data Fig. 3 [file 44319_2024_241_MOESM4_ESM.zip › Figure3/3B/lpp>pgk-i.JPG]

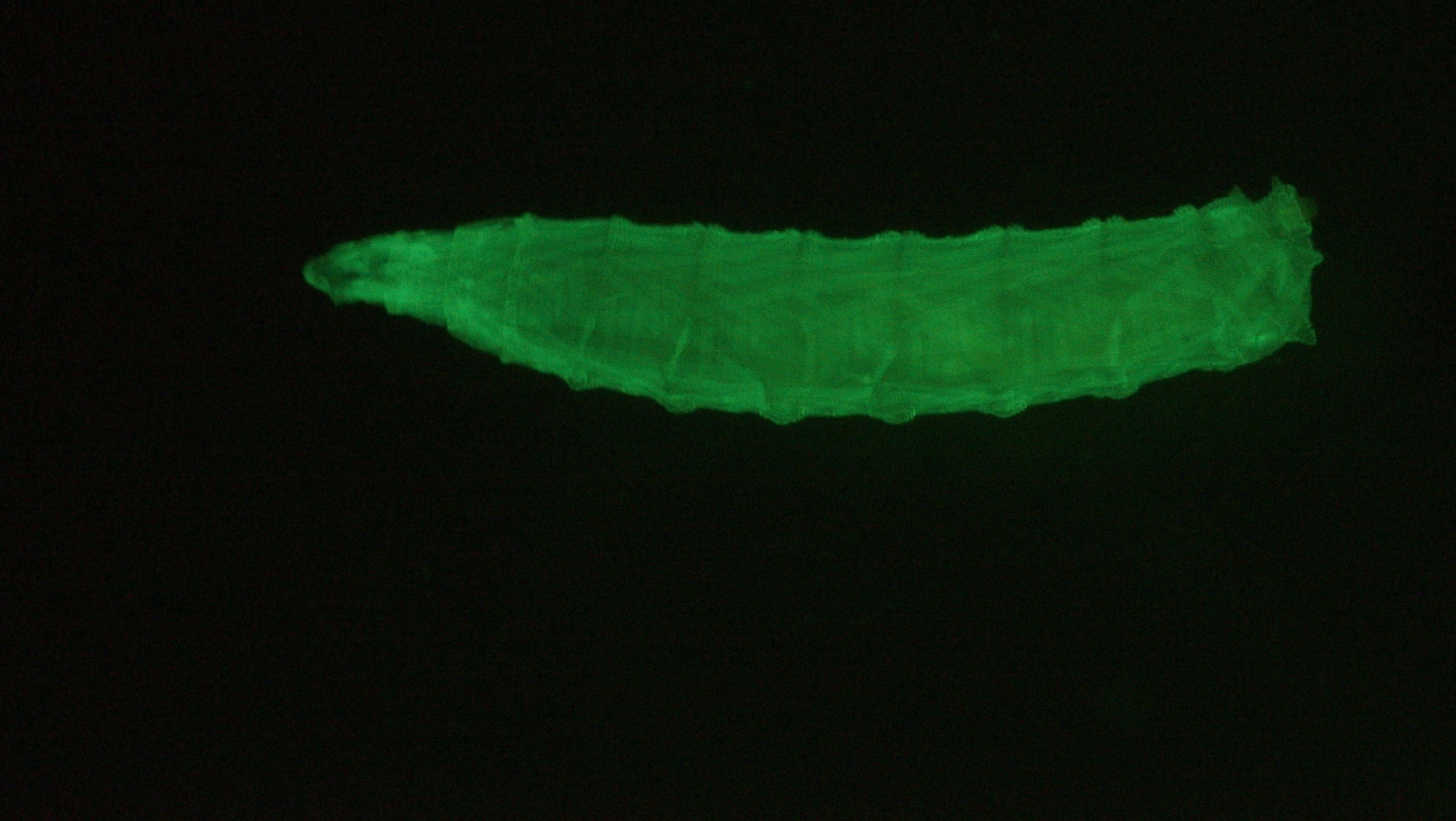

Supplement: Supplementary file 4 — Source data Fig. 3 [file 44319_2024_241_MOESM4_ESM.zip › Figure3/3B/lpp>pglym78-i.JPG]

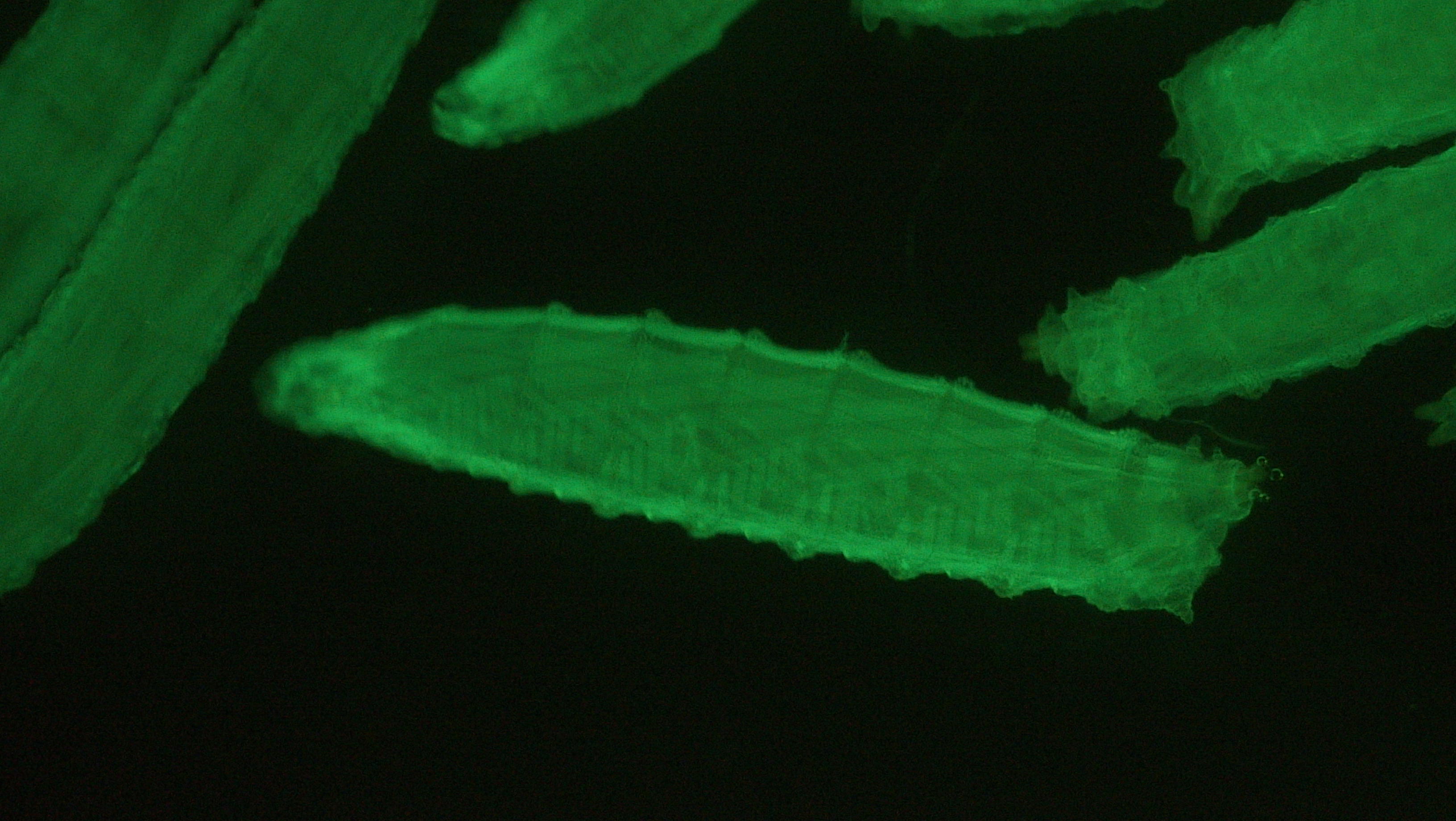

Supplement: Supplementary file 4 — Source data Fig. 3 [file 44319_2024_241_MOESM4_ESM.zip › Figure3/3B/lpp>w-i.JPG]

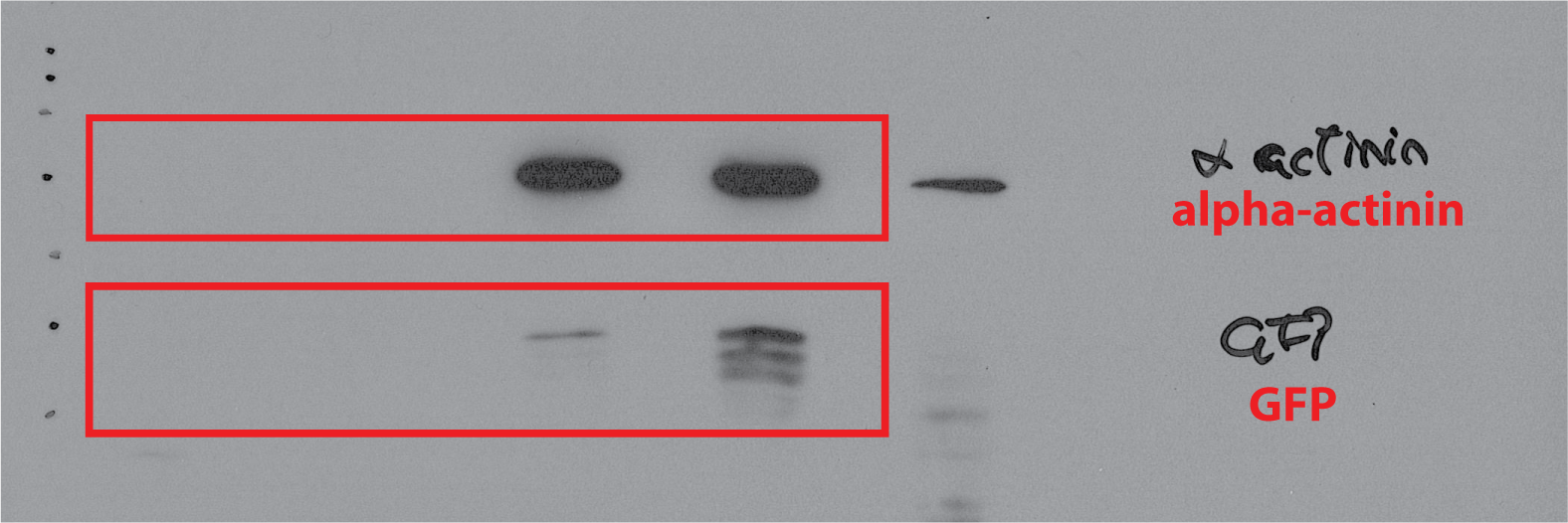

Supplement: Supplementary file 4 — Source data Fig. 3 [file 44319_2024_241_MOESM4_ESM.zip › Figure3/3D/WB a-actinin & GFP.tif]

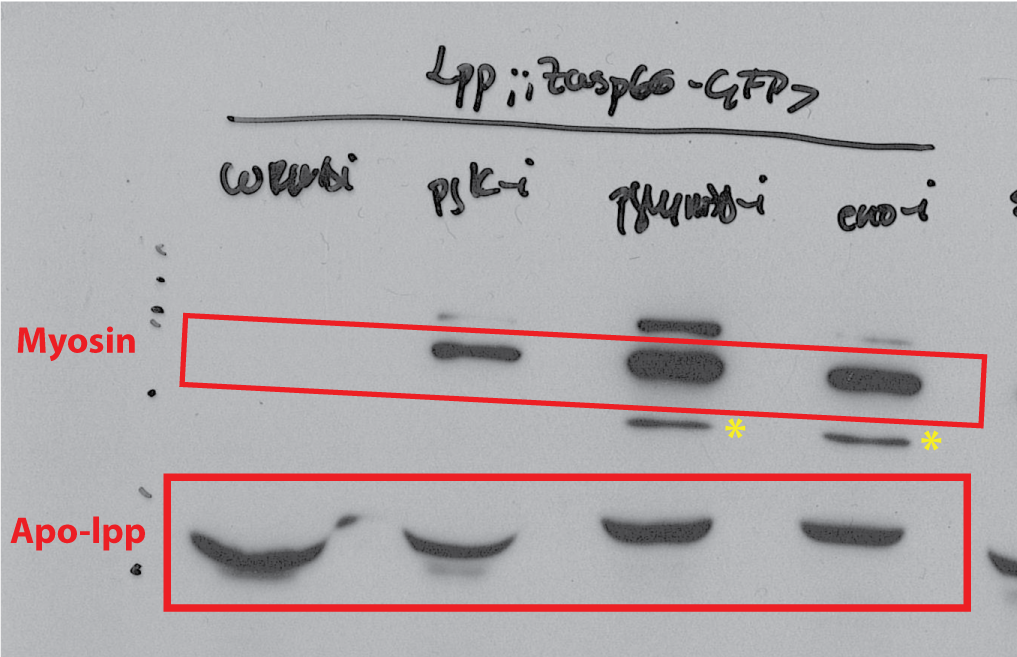

Supplement: Supplementary file 4 — Source data Fig. 3 [file 44319_2024_241_MOESM4_ESM.zip › Figure3/3D/WB Myosin & Apo-Lpp.tif]

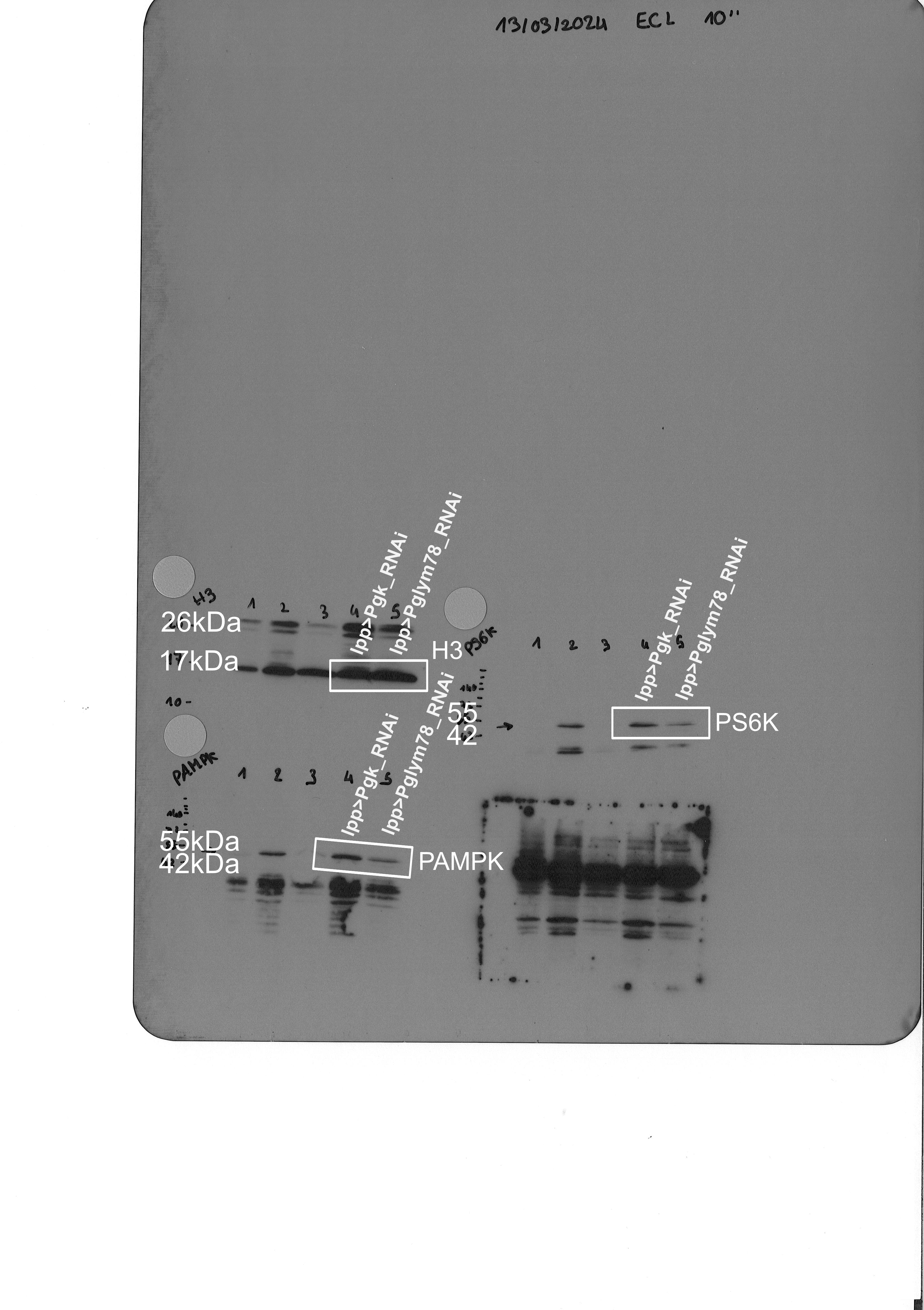

Supplement: Supplementary file 5 — Source data Fig. 4 [file 44319_2024_241_MOESM5_ESM.zip › Figure4/4B/PAMPK_H3_PS6K.jpg]

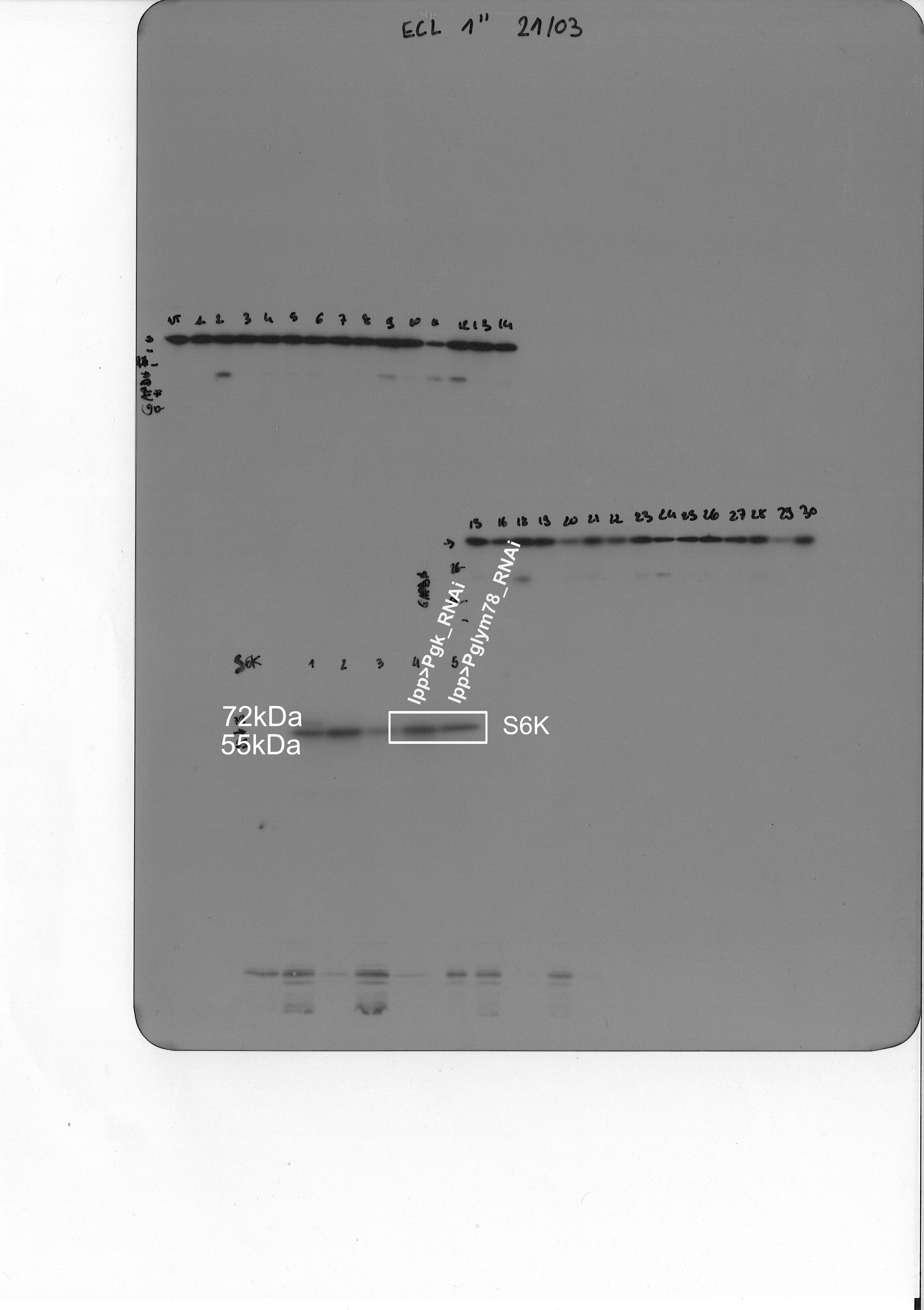

Supplement: Supplementary file 5 — Source data Fig. 4 [file 44319_2024_241_MOESM5_ESM.zip › Figure4/4B/S6K.jpg]

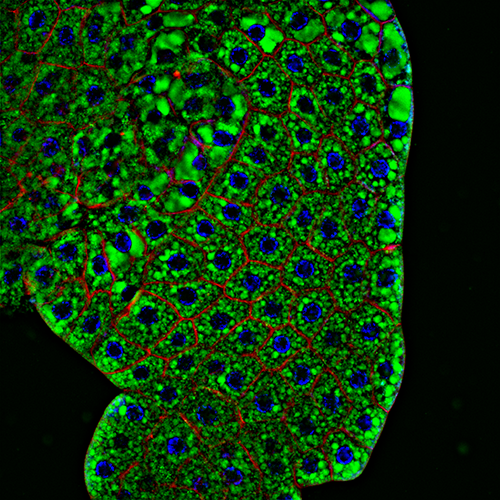

Supplement: Supplementary file 5 — Source data Fig. 4 [file 44319_2024_241_MOESM5_ESM.zip › Figure4/4E/lpp>pglym78-i,Reptor-i.tif]

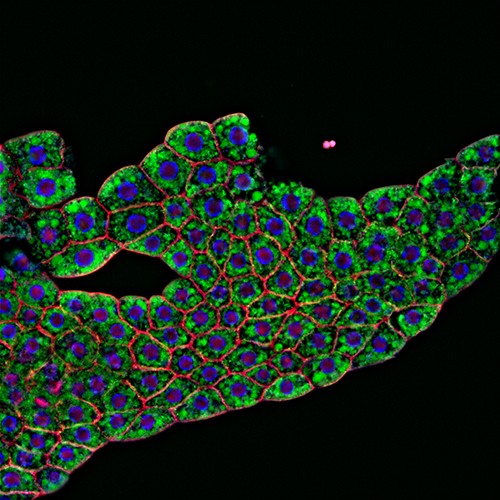

Supplement: Supplementary file 5 — Source data Fig. 4 [file 44319_2024_241_MOESM5_ESM.zip › Figure4/4E/lpp>pglym78-i,w-i.tif]

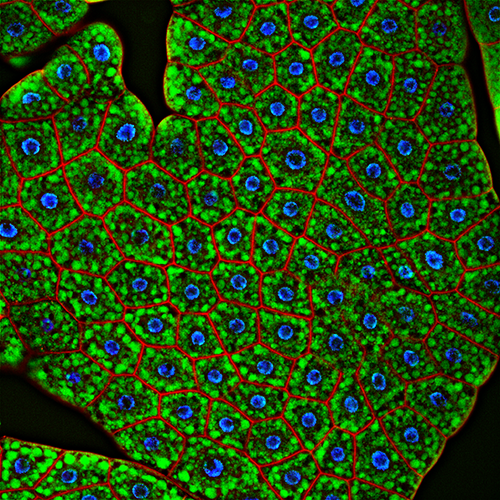

Supplement: Supplementary file 5 — Source data Fig. 4 [file 44319_2024_241_MOESM5_ESM.zip › Figure4/4E/lpp>xw-i.tif]

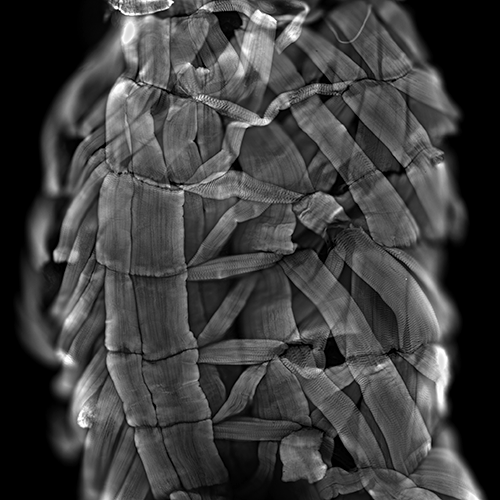

Supplement: Supplementary file 5 — Source data Fig. 4 [file 44319_2024_241_MOESM5_ESM.zip › Figure4/4F/lpp>pglym78-i,Reptor-i.tif]

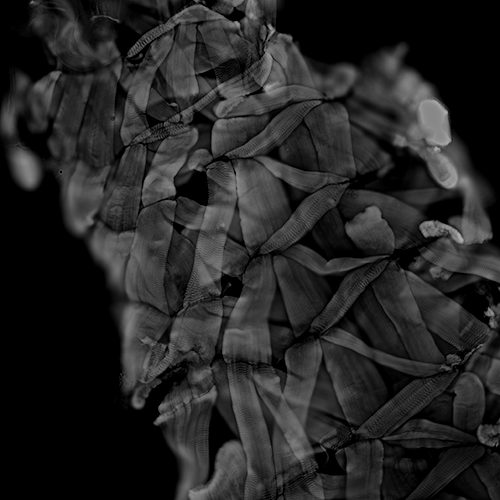

Supplement: Supplementary file 5 — Source data Fig. 4 [file 44319_2024_241_MOESM5_ESM.zip › Figure4/4F/lpp>pglym78-i,w-ii.tif]

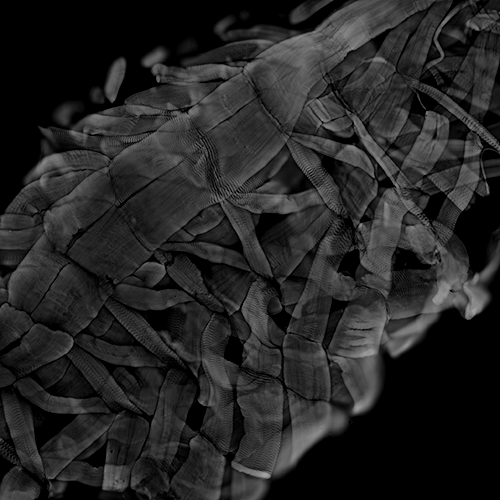

Supplement: Supplementary file 5 — Source data Fig. 4 [file 44319_2024_241_MOESM5_ESM.zip › Figure4/4F/lpp>w-i.tif]

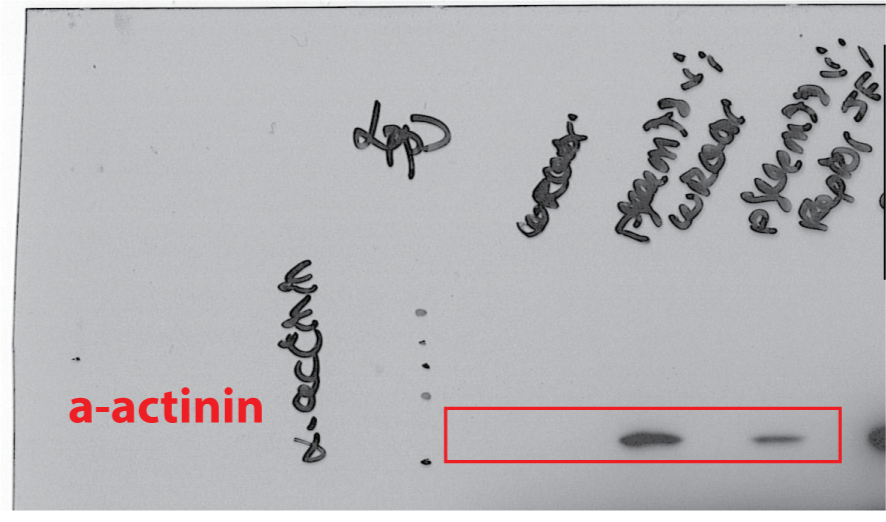

Supplement: Supplementary file 5 — Source data Fig. 4 [file 44319_2024_241_MOESM5_ESM.zip › Figure4/4I/WB a-actinin.tif]

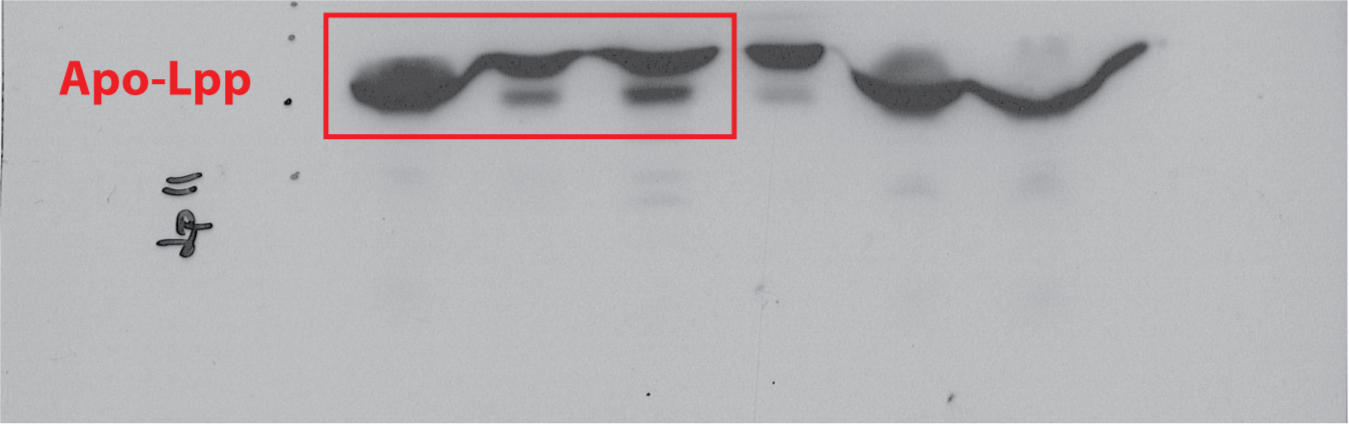

Supplement: Supplementary file 5 — Source data Fig. 4 [file 44319_2024_241_MOESM5_ESM.zip › Figure4/4I/WB Apo_lpp.tif]

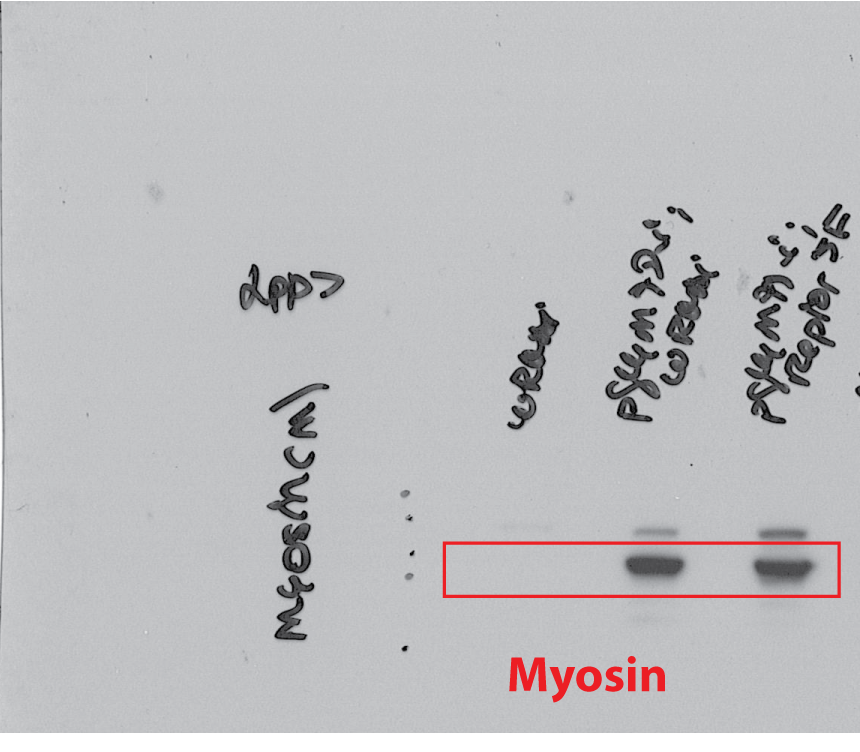

Supplement: Supplementary file 5 — Source data Fig. 4 [file 44319_2024_241_MOESM5_ESM.zip › Figure4/4I/WB Myosin.tif]

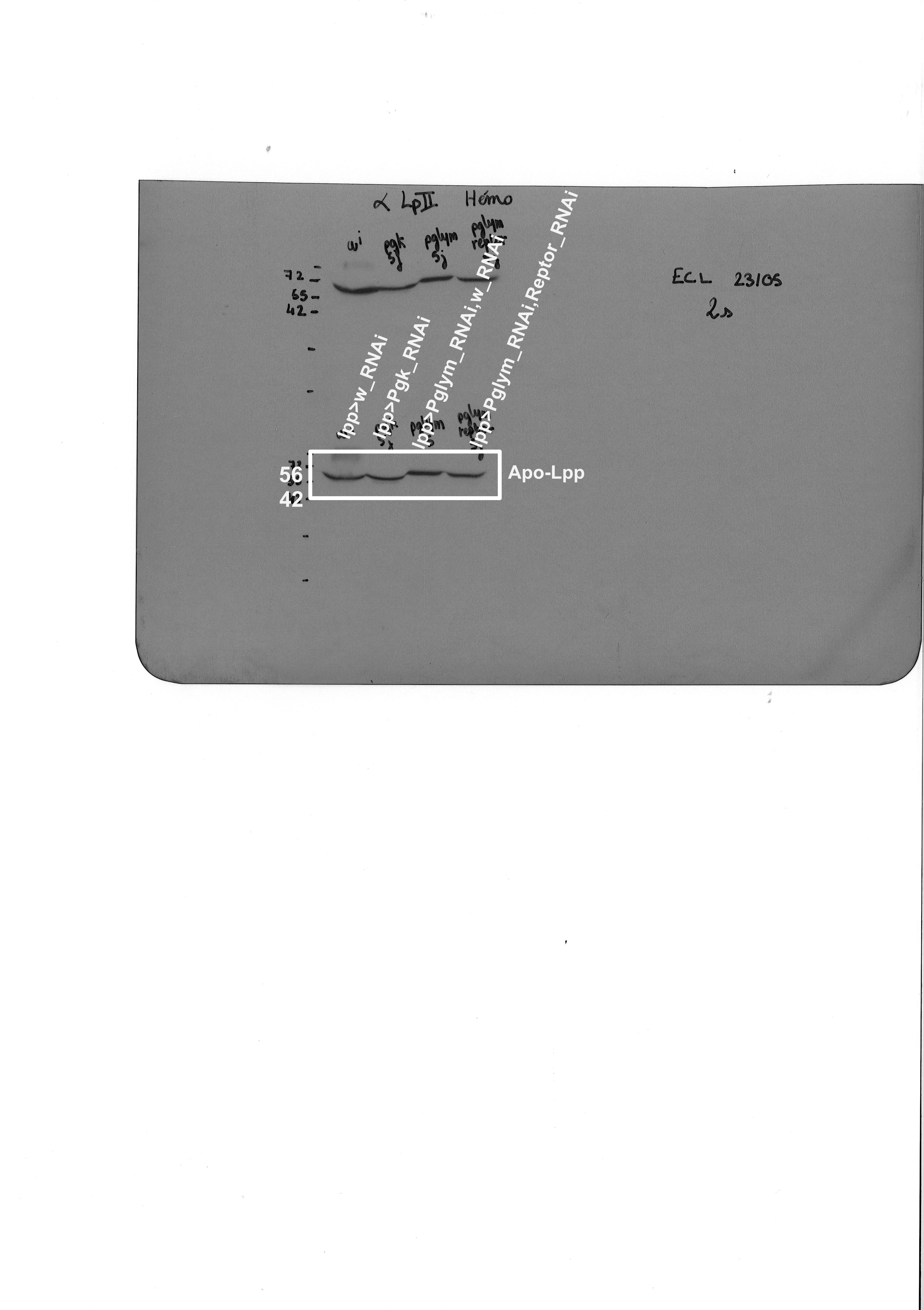

Supplement: Supplementary file 6 — Source data Fig. 5 [file 44319_2024_241_MOESM6_ESM.zip › Figure5/5B/WB_ApoLpp.jpg]

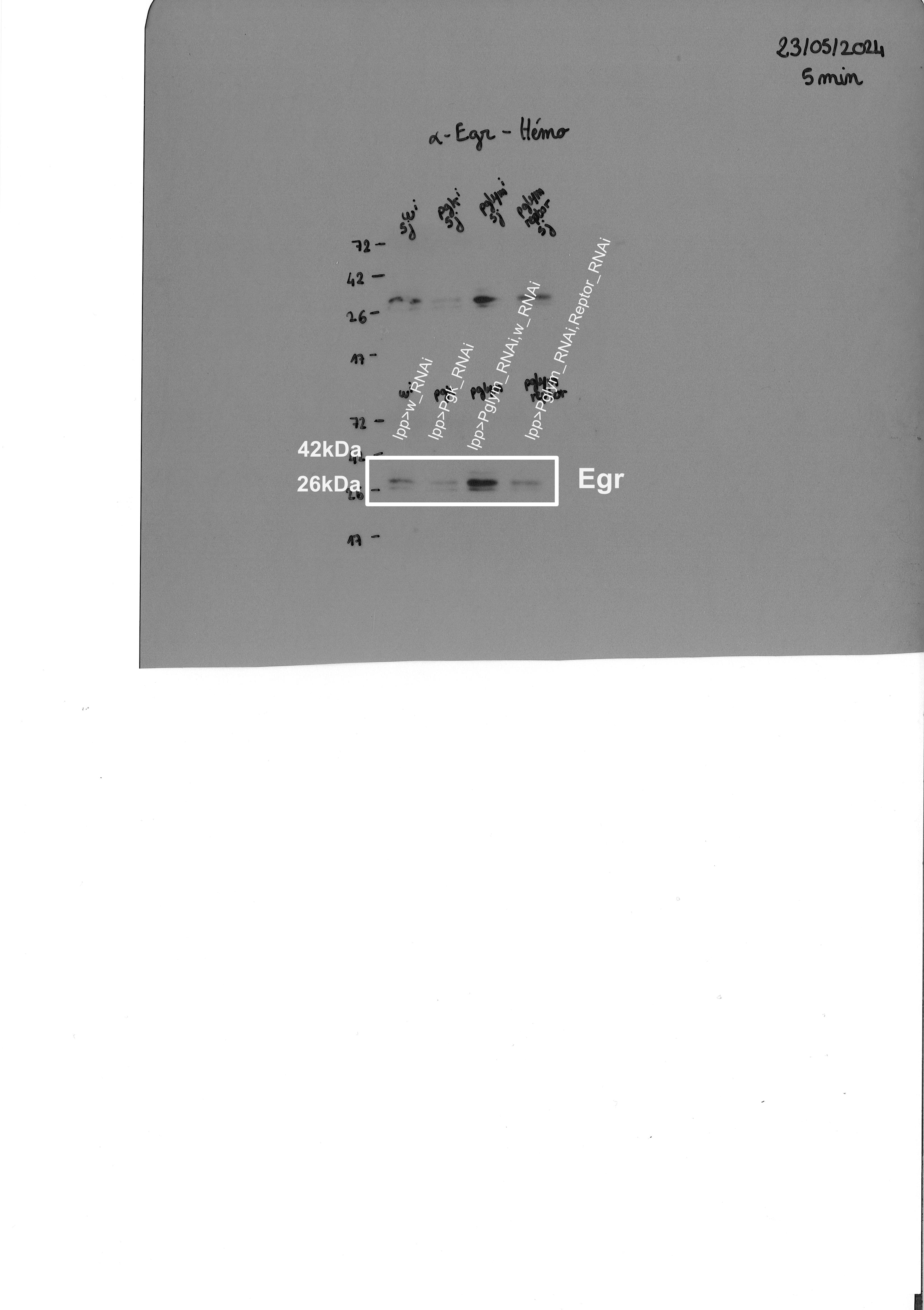

Supplement: Supplementary file 6 — Source data Fig. 5 [file 44319_2024_241_MOESM6_ESM.zip › Figure5/5B/WB_Egr.jpg]

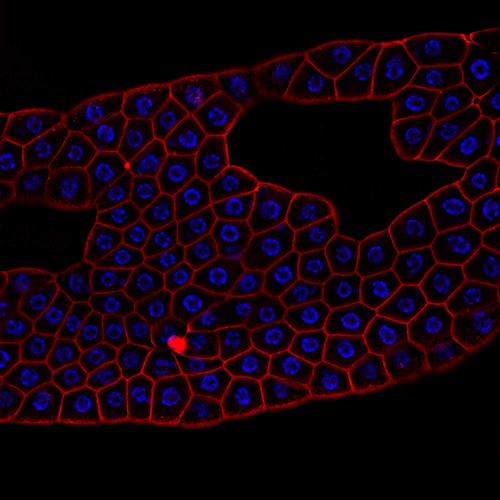

Supplement: Supplementary file 6 — Source data Fig. 5 [file 44319_2024_241_MOESM6_ESM.zip › Figure5/5C/Lpp>pglym-i; w-i.tif]

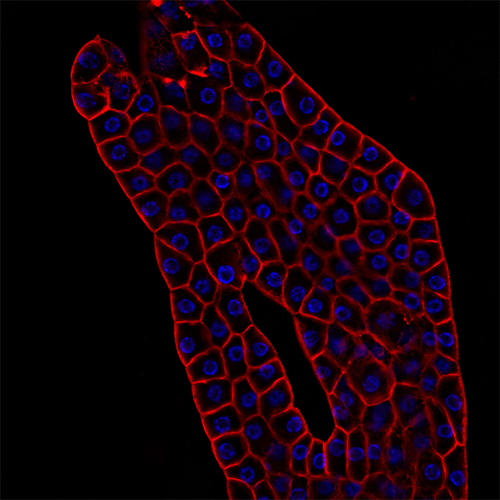

Supplement: Supplementary file 6 — Source data Fig. 5 [file 44319_2024_241_MOESM6_ESM.zip › Figure5/5C/Lpp>pglym-i;tace-i.tif]

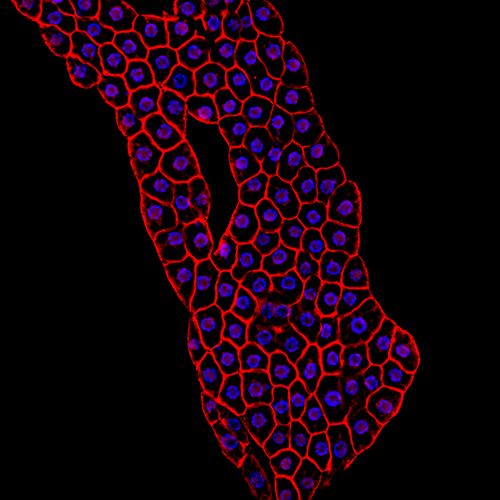

Supplement: Supplementary file 6 — Source data Fig. 5 [file 44319_2024_241_MOESM6_ESM.zip › Figure5/5C/Lpp>pglym78-i,egrIR.tif]

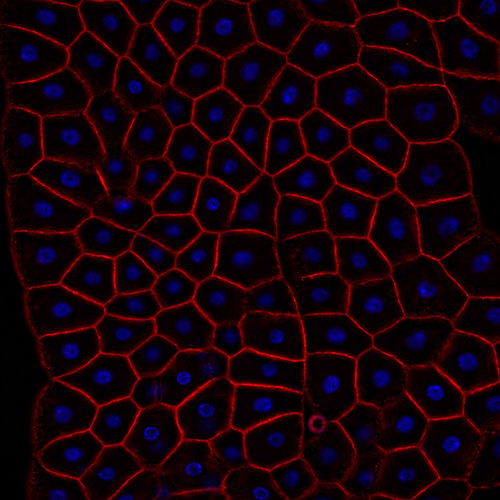

Supplement: Supplementary file 6 — Source data Fig. 5 [file 44319_2024_241_MOESM6_ESM.zip › Figure5/5C/Lpp>w-i.tif]

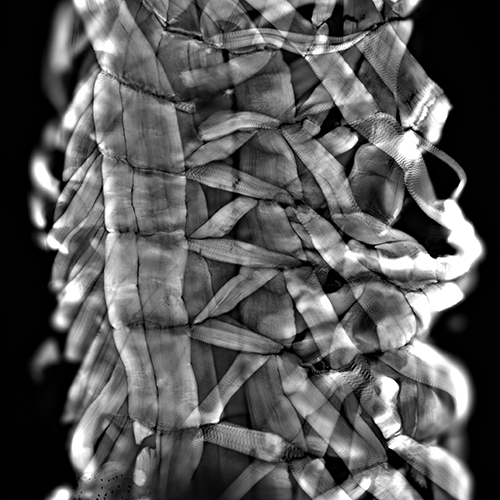

Supplement: Supplementary file 6 — Source data Fig. 5 [file 44319_2024_241_MOESM6_ESM.zip › Figure5/5D/Lpp>pglym78-i, egrIR.tif]

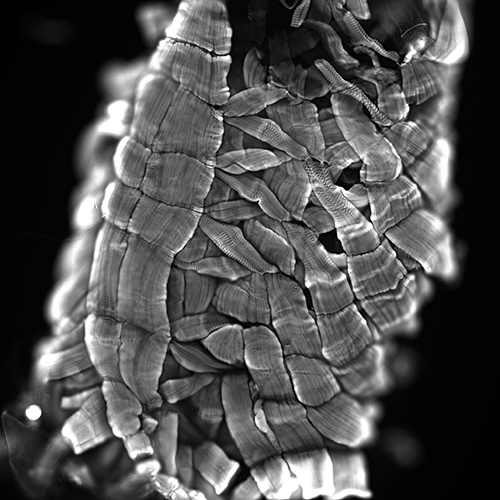

Supplement: Supplementary file 6 — Source data Fig. 5 [file 44319_2024_241_MOESM6_ESM.zip › Figure5/5D/Lpp>pglym78-i,tace-i.tif]

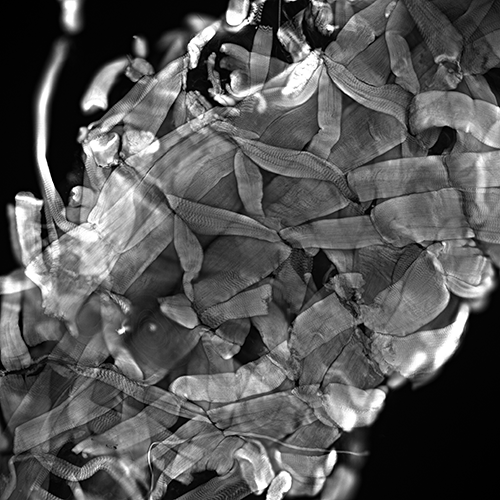

Supplement: Supplementary file 6 — Source data Fig. 5 [file 44319_2024_241_MOESM6_ESM.zip › Figure5/5D/Lpp>pglym78-i,w-i.tif]

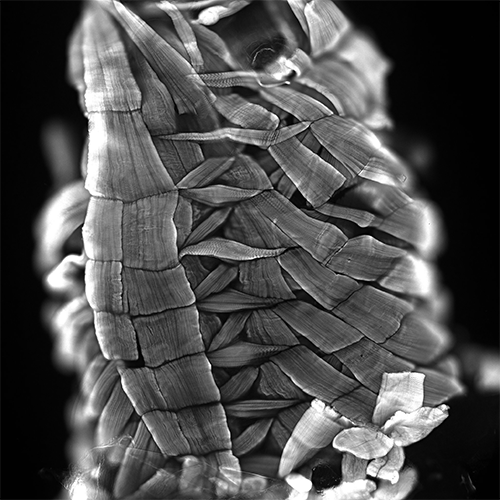

Supplement: Supplementary file 6 — Source data Fig. 5 [file 44319_2024_241_MOESM6_ESM.zip › Figure5/5D/Lpp>w-i.tif]

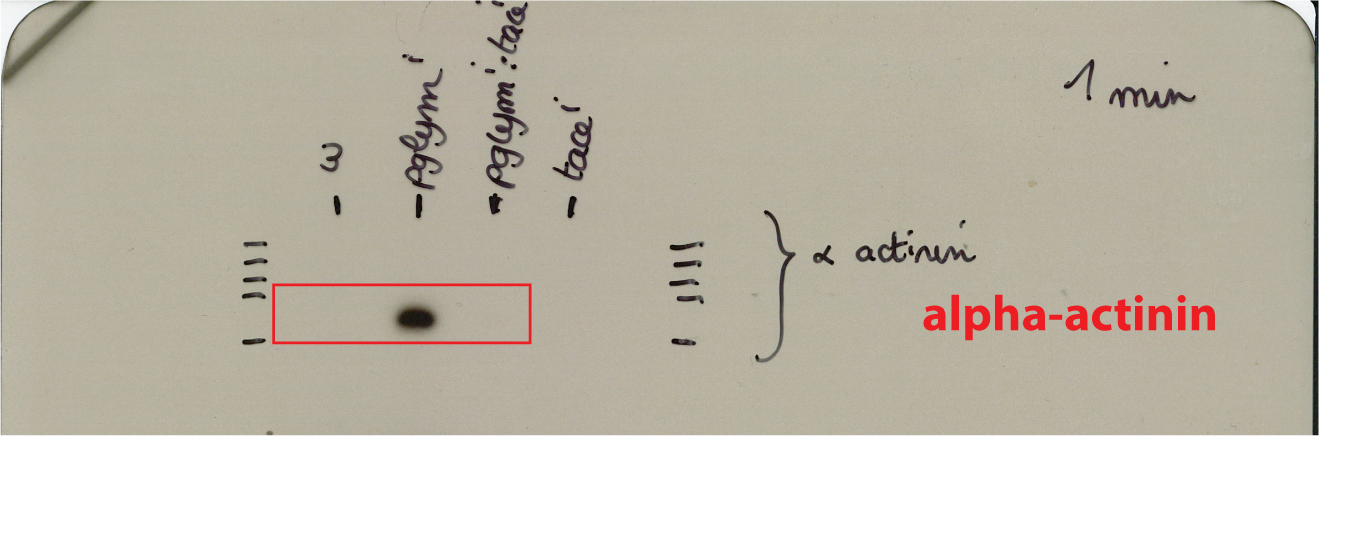

Supplement: Supplementary file 6 — Source data Fig. 5 [file 44319_2024_241_MOESM6_ESM.zip › Figure5/5G/WB a-actinin.tif]

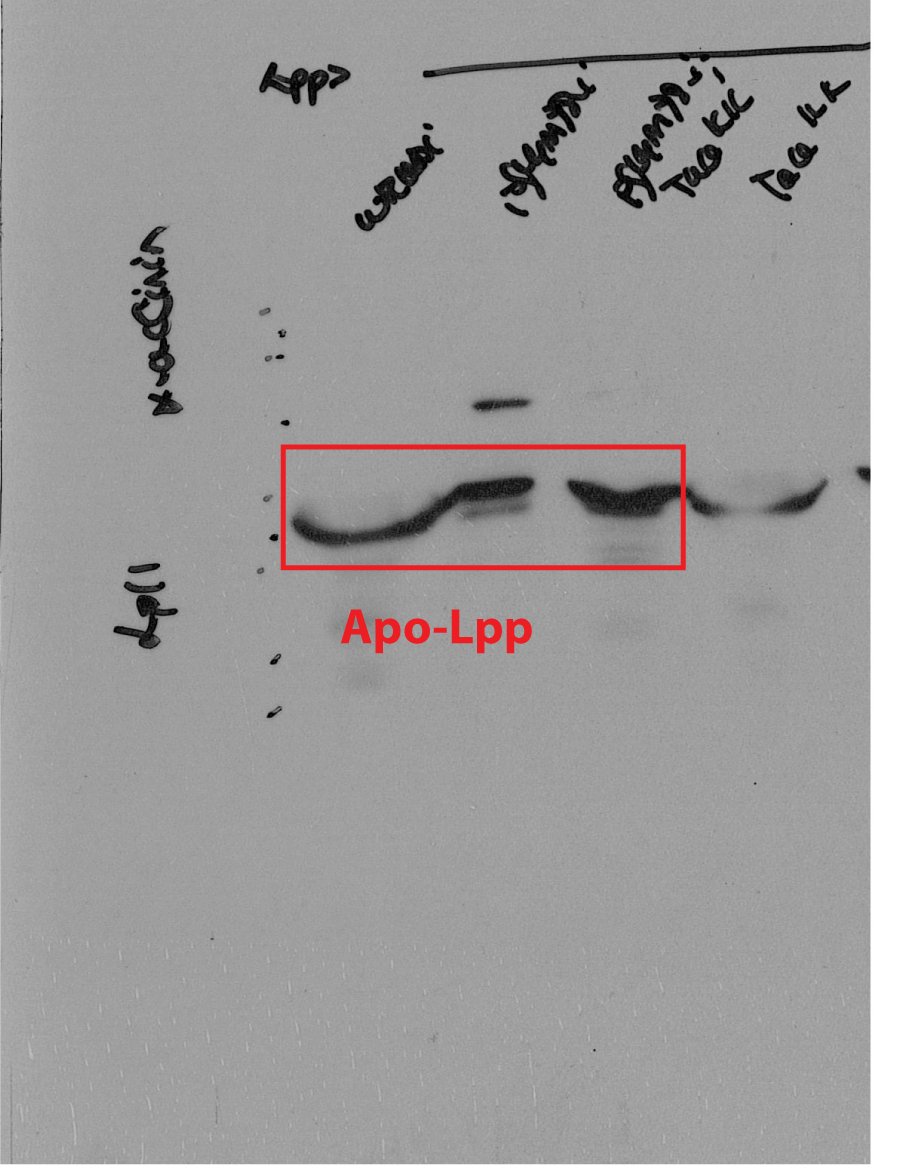

Supplement: Supplementary file 6 — Source data Fig. 5 [file 44319_2024_241_MOESM6_ESM.zip › Figure5/5G/WB Apo-Lpp.tif]

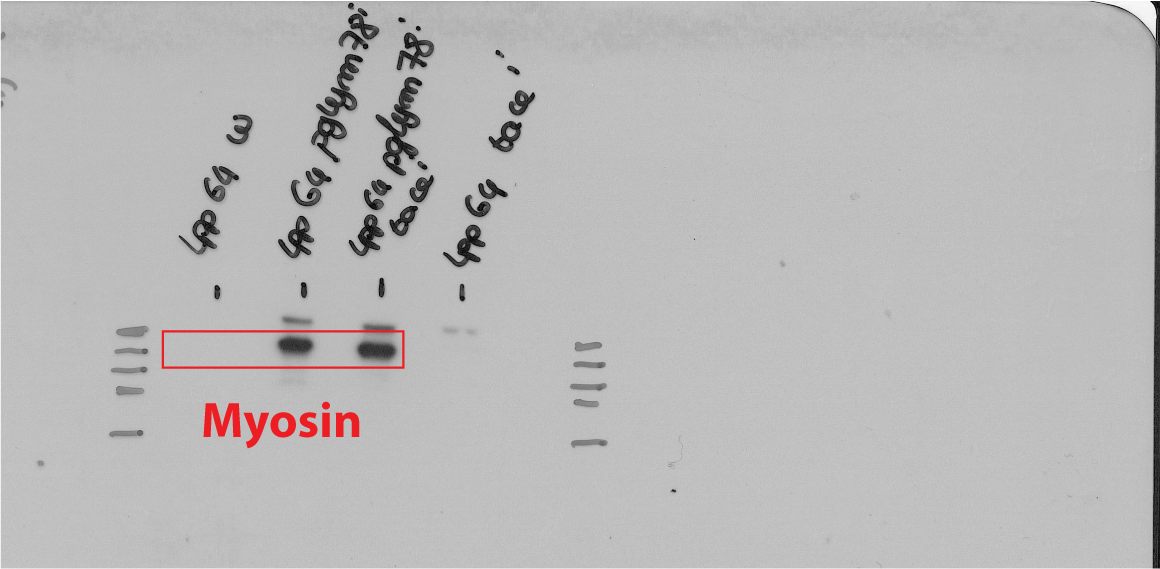

Supplement: Supplementary file 6 — Source data Fig. 5 [file 44319_2024_241_MOESM6_ESM.zip › Figure5/5G/WB Myosin.tif]

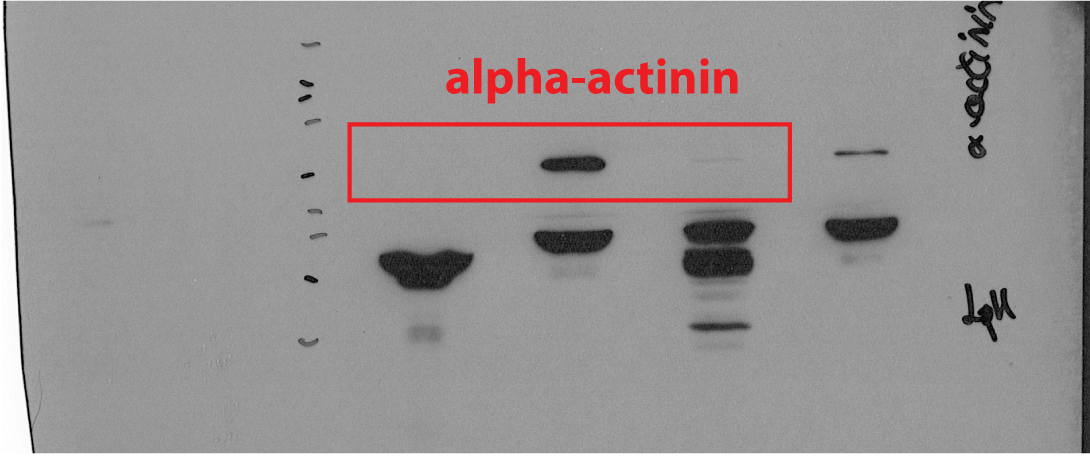

Supplement: Supplementary file 6 — Source data Fig. 5 [file 44319_2024_241_MOESM6_ESM.zip › Figure5/5H/WB a-actinin.tif]

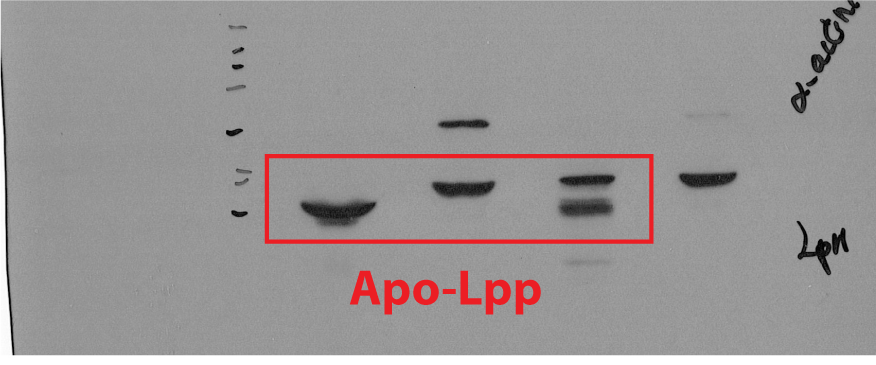

Supplement: Supplementary file 6 — Source data Fig. 5 [file 44319_2024_241_MOESM6_ESM.zip › Figure5/5H/WB apo-Lpp.tif]

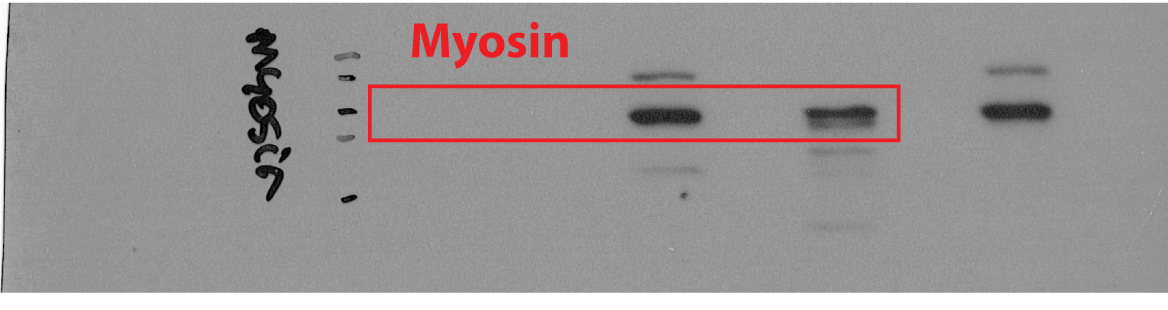

Supplement: Supplementary file 6 — Source data Fig. 5 [file 44319_2024_241_MOESM6_ESM.zip › Figure5/5H/WB myosin.tif]

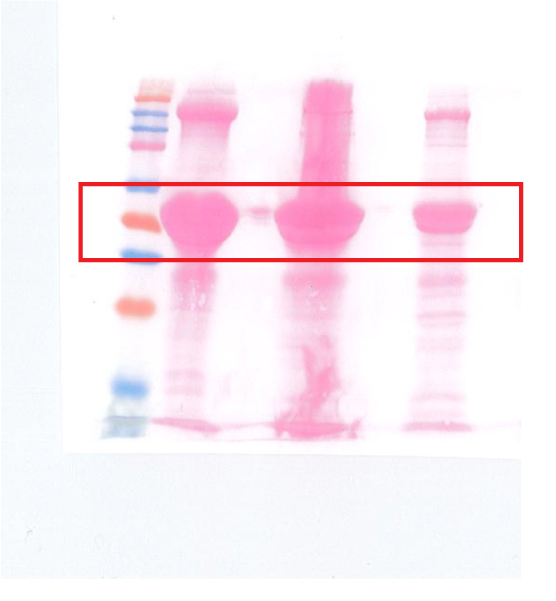

Supplement: Supplementary file 6 — Source data Fig. 5 [file 44319_2024_241_MOESM6_ESM.zip › Figure5/5I/Ponceau.tif]

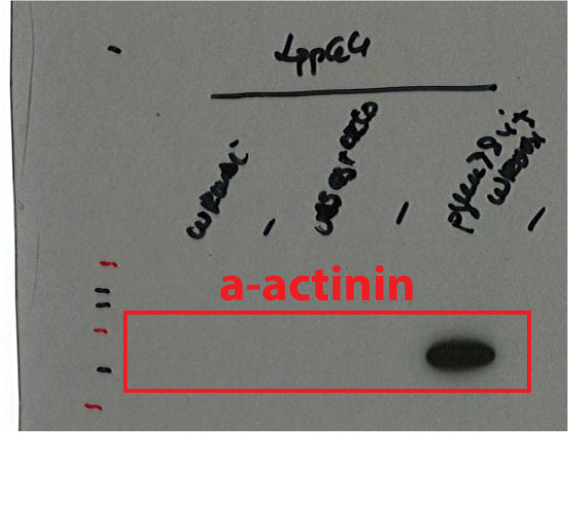

Supplement: Supplementary file 6 — Source data Fig. 5 [file 44319_2024_241_MOESM6_ESM.zip › Figure5/5I/WB a-actinin.tif]

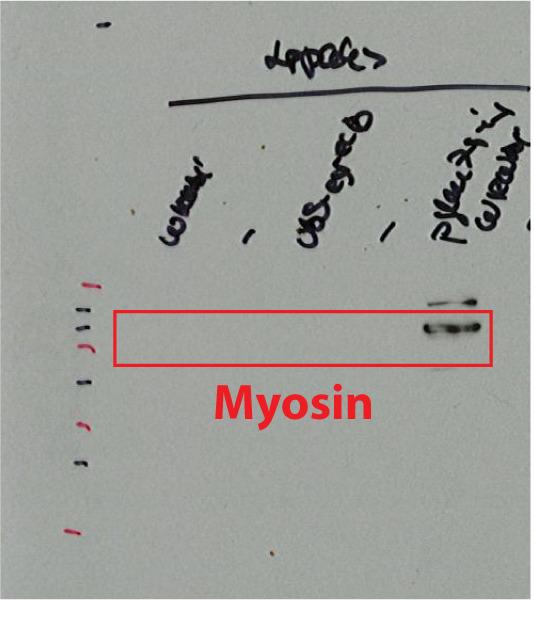

Supplement: Supplementary file 6 — Source data Fig. 5 [file 44319_2024_241_MOESM6_ESM.zip › Figure5/5I/WB myosin.tif]

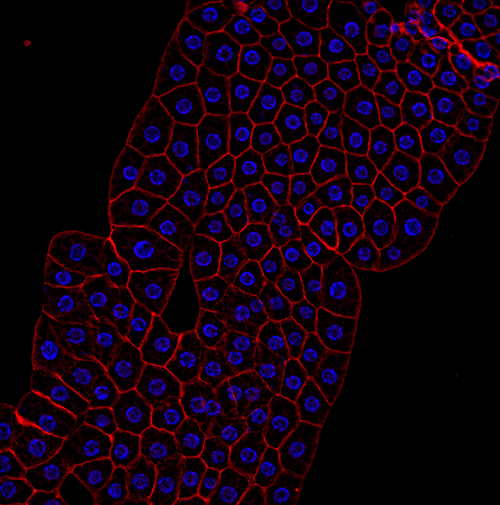

Supplement: Supplementary file 7 — Source data Fig. 6 [file 44319_2024_241_MOESM7_ESM.zip › Figure6/6C/Lpp>pglym78-i,w-i.tif]

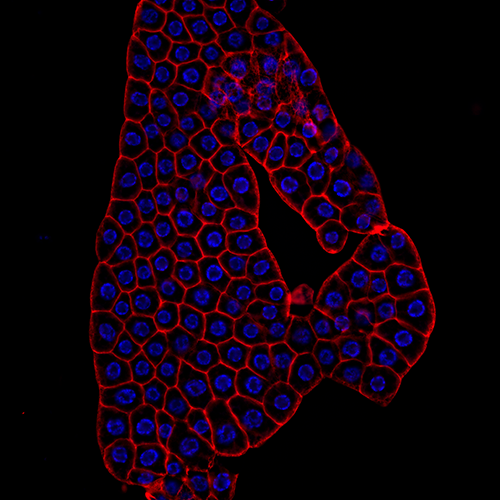

Supplement: Supplementary file 7 — Source data Fig. 6 [file 44319_2024_241_MOESM7_ESM.zip › Figure6/6C/Lpp>pglym78-i;ImpL2-i.tif]

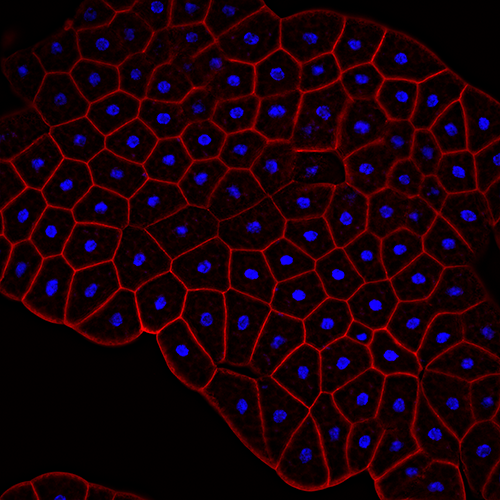

Supplement: Supplementary file 7 — Source data Fig. 6 [file 44319_2024_241_MOESM7_ESM.zip › Figure6/6C/Lpp>w-i.tif]

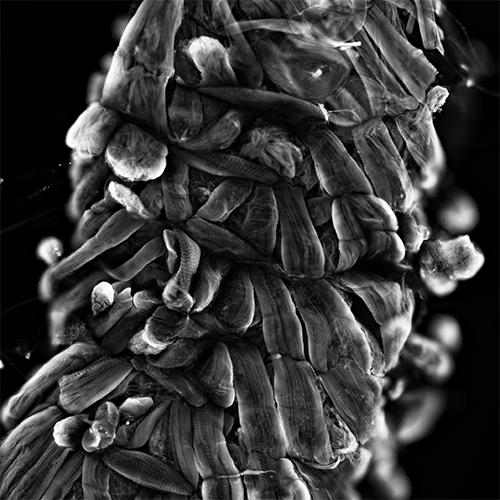

Supplement: Supplementary file 7 — Source data Fig. 6 [file 44319_2024_241_MOESM7_ESM.zip › Figure6/6D/Lpp>pglym78-i, w-i.tif]

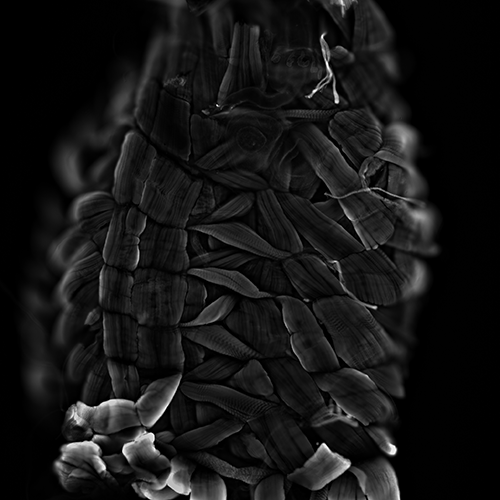

Supplement: Supplementary file 7 — Source data Fig. 6 [file 44319_2024_241_MOESM7_ESM.zip › Figure6/6D/Lpp>pglym78-i,ImpL2-i.tif]

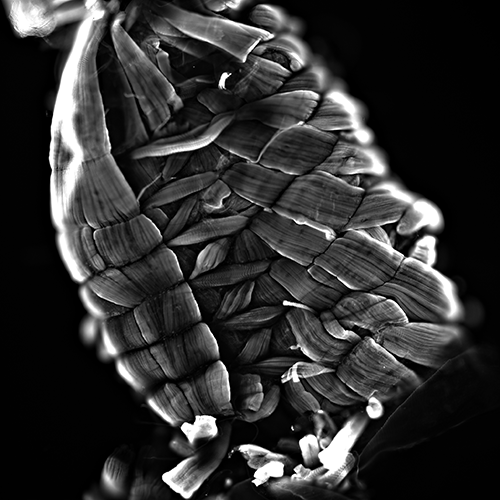

Supplement: Supplementary file 7 — Source data Fig. 6 [file 44319_2024_241_MOESM7_ESM.zip › Figure6/6D/Lpp>w-i.tif]

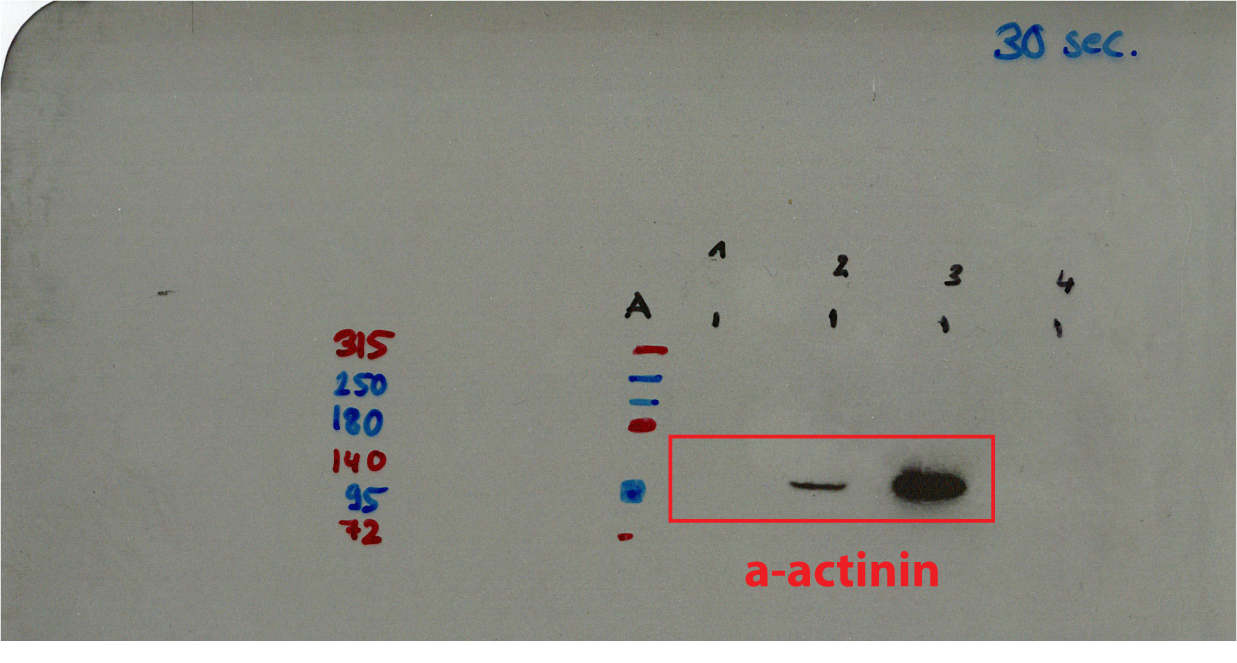

Supplement: Supplementary file 7 — Source data Fig. 6 [file 44319_2024_241_MOESM7_ESM.zip › Figure6/6G/WB a-actinin.tif]

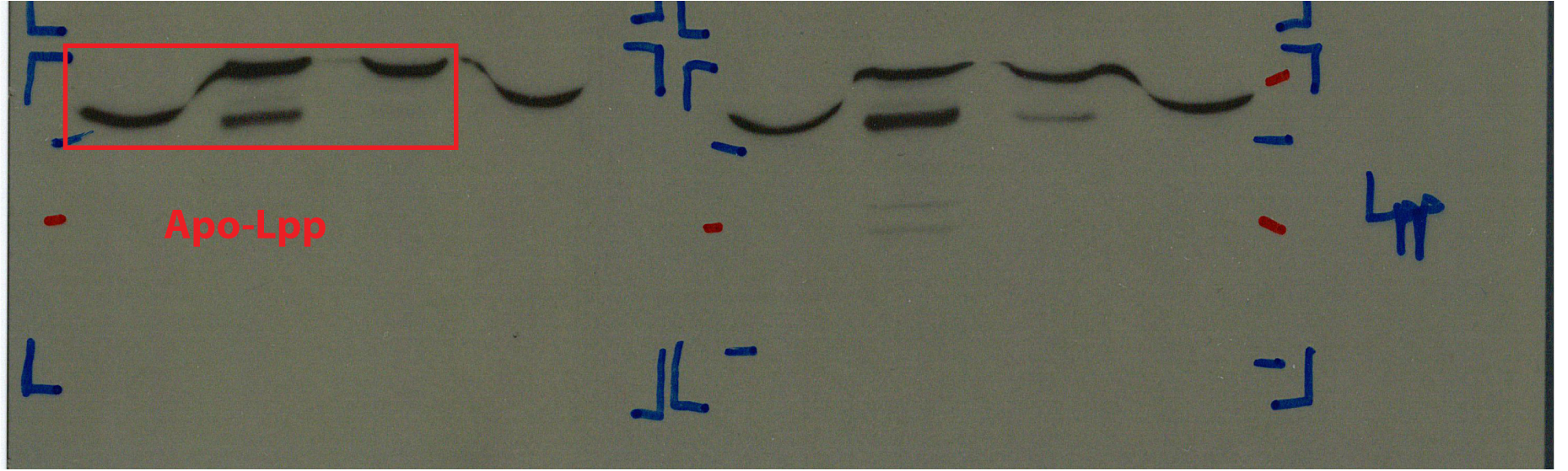

Supplement: Supplementary file 7 — Source data Fig. 6 [file 44319_2024_241_MOESM7_ESM.zip › Figure6/6G/WB Apo-Lpp.tif]

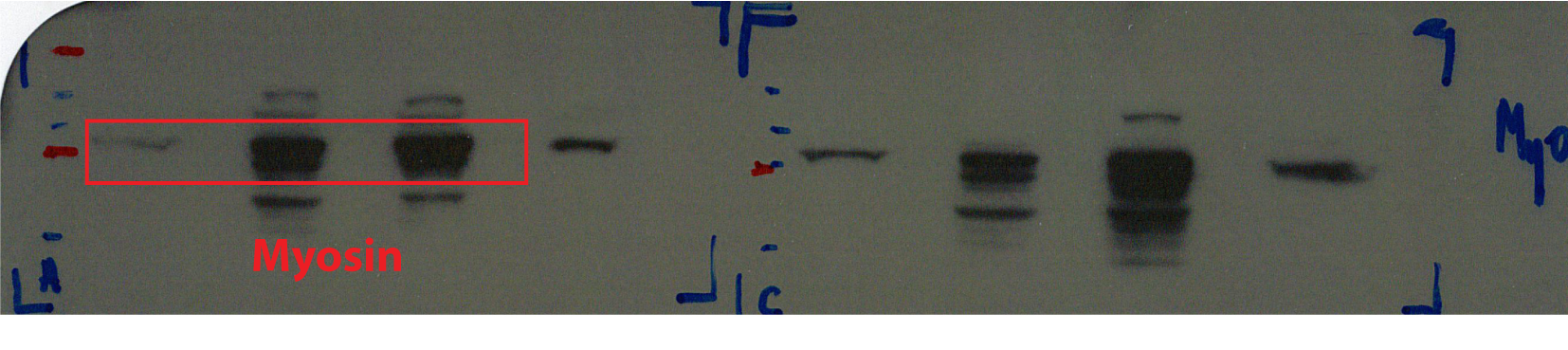

Supplement: Supplementary file 7 — Source data Fig. 6 [file 44319_2024_241_MOESM7_ESM.zip › Figure6/6G/WB Myosin.tif]
