# Supplementary material for: Fat body glycolysis defects inhibit mTOR and promote distant muscle disorganization through TNF-α/egr and ImpL2 signaling in Drosophila larvae
Source: EMBO Rep. 2024 Sep 9;25(10):4410–32. doi: 10.1038/s44319-024-00241-3 (PMC11467327; doi:10.1038/s44319-024-00241-3)
Supplement: Supplementary file 9 — Expanded View Figures [file 44319_2024_241_MOESM9_ESM.pdf]

Expanded View Figures

Figure EV1. Fat body atrophy after *Pglym78* knock-down (related to Fig. 1).

(**A**) Evaluation of the knock-down efficiency of the different RNAi used monitored by qRT-PCR. Reference gene for normalization: *rp49/RpL32*. Biological replicates $n = 3$. Error bars show the standard error of the mean (sem). One-way ANOVA statistical test, ****$p < 0.0001$. (**B**) Fat body staining of the indicated genotypes showing nuclei (DAPI, blue) and cell cortex (F-actin, red or white); white bar 50 µm. (**C**) Quantification of average cell size from images shown in (**B**), and represented has box plots where the whiskers represent the maxima and minima experimental points, the boxes represent the 25 and 75% percentiles, and where center line is the median. Biological replicates $n = 7$–11. Mann–Whitney test, **$p = 0.0043$. (**D**) Glycogen content in the adipose tissue after *Pglym78* knock-down compared to control monitored by periodic shift acid staining. White and black bars 200 µm. (**E**, **F**) Circulating levels of trehalose in the hemolymph (**D** left), of glycogen in larvae (**D** right), and of triglycerides in larvae (TAG; **E**) after *Pglym78* knock-down in fat body cells compared to controls. Error bars show the standard error of the mean (sem). Biological replicates $n = 3$ to 5. One-way ANOVA statistical test, ns not significant. $p > 0.05$ (**E**), or Mann–Whitney test, *$p = 0.0260$ (**F**). (**G**, **H**) Quantification of ATP (**G**) and pyruvate (**H**) content in dissected fat bodies normalized to total nucleic acid content. Biological triplicates. Error bars show the standard error of the mean (sem). Mann–Whitney test, **$p = 0.0079$ (**G**) or one-way ANOVA statistical test, *$p = 0.0374$, ns not significant (**H**). Source data are available online for this figure.

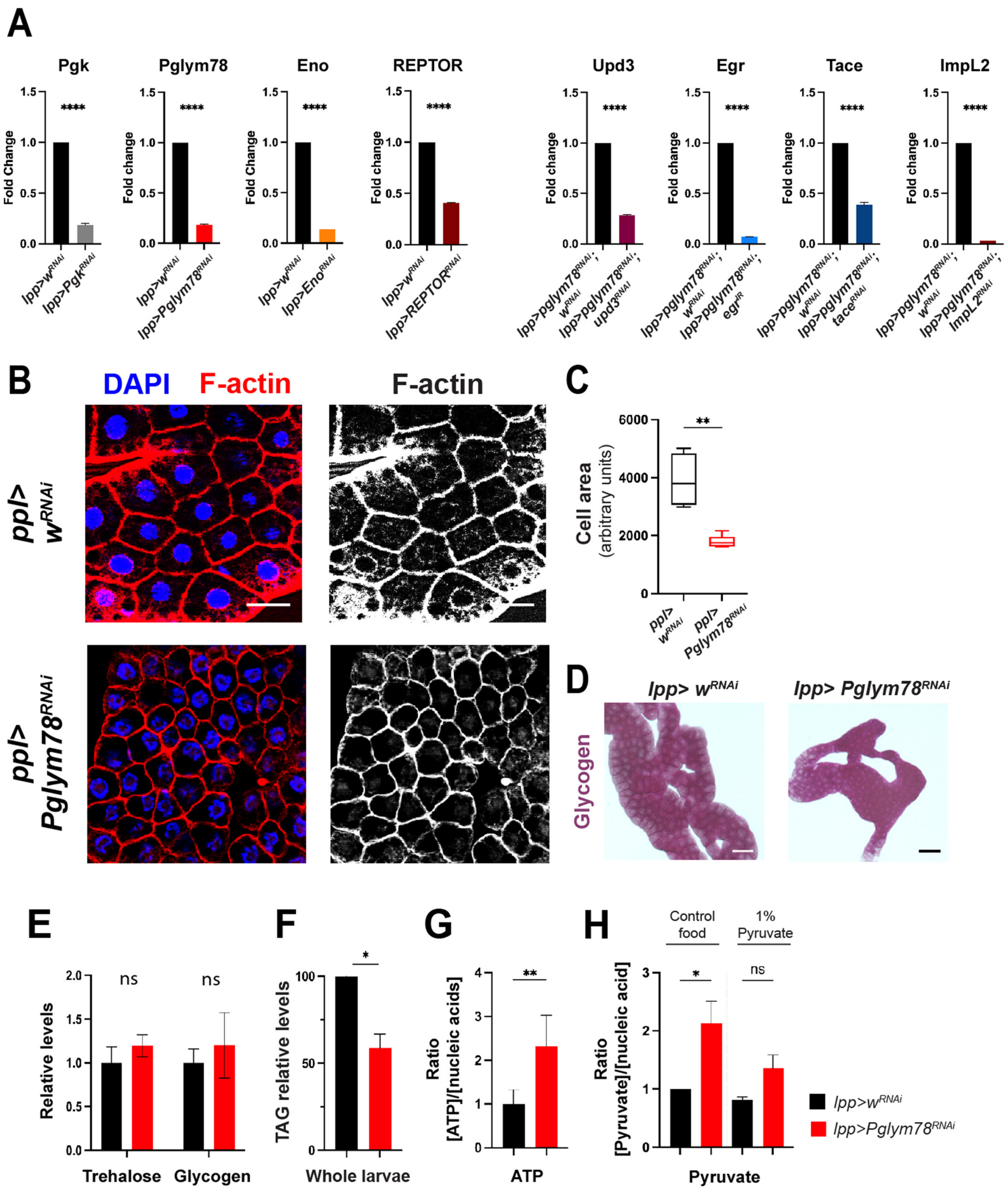
A
Pgk
Pglym78
Eno
REPTOR
Upd3
Egr
Tace
ImpL2
Fold Change
Fold change
1.5
1.0
0.5
0.0
****
lpp>w^RNAi
lpp>Pgk^RNAi
lpp>Pglym78^RNAi
lpp>Eno^RNAi
lpp>REPTOR^RNAi
lpp>pglym78^RNAi; w^RNAi
lpp>pglym78^RNAi; upd3^RNAi
lpp>pglym78^RNAi; egr^IR
lpp>pglym78^RNAi; tace^RNAi
lpp>pglym78^RNAi; ImpL2^RNAi
B
DAPI F-actin
F-actin
ppl> w^RNAi
ppl> Pglym78^RNAi
C
Cell area (arbitrary units)
6000
4000
2000
0
**
ppl> w^RNAi
ppl> Pglym78^RNAi
D
lpp> w^RNAi
lpp> Pglym78^RNAi
Glycogen
E
Relative levels
2.0
1.5
1.0
0.5
0.0
ns
ns
Trehalose
Glycogen
F
TAG relative levels
100
50
0
*
Whole larvae
G
Ratio [ATP]/[nucleic acids]
4
3
2
1
0
**
ATP
H
Control food
1% Pyruvate
Ratio [Pyruvate]/[nucleic acid]
3
2
1
0
*
ns
Pyruvate
lpp>w^RNAi
lpp>Pglym78^RNAi

 © The Author(s)

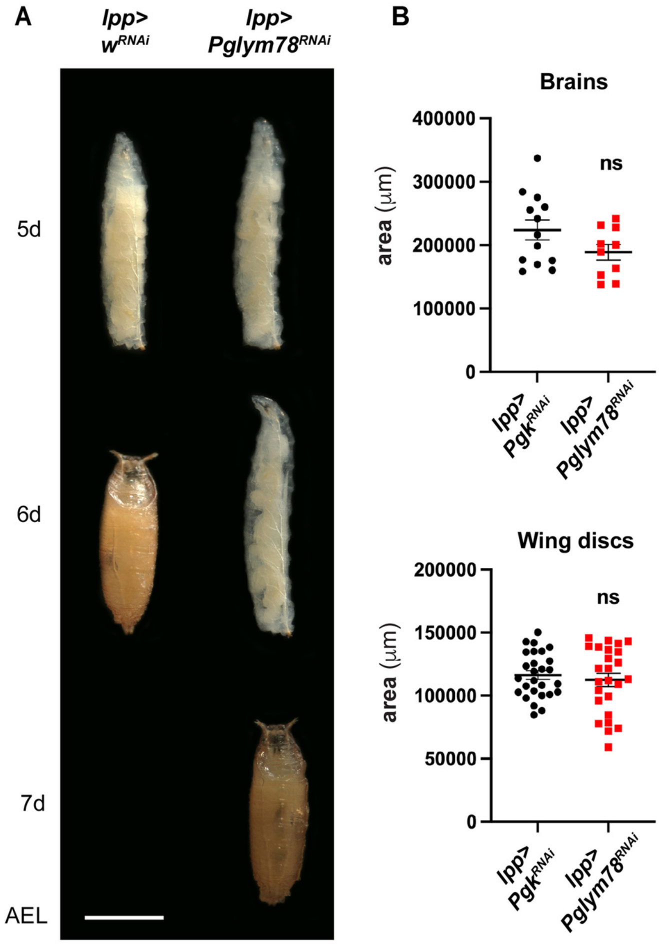

Figure EV2. Developmental delay after fat body *Pglym78* knock down (related to Fig. 2).

(**A**) Images of representative larvae and pupae of *lppGal4> Pglym78-RNAi* and *lppGal4 > w-RNAi* controls at the 5, 6, and 7 days (d) after egg laying (ael); white bar 1 mm. (**B**) Size of brains and wing disks expressed in arbitrary units (pixels) in *Pglym78-RNAi* animals compared to *Pgk-RNAi* controls at 6 days ael. For brains, $n = 13$ and 10, for disks, $n = 27$ and 24. Mann–Whitney test. ns not significant. Source data are available online for this figure.

© The Author(s)

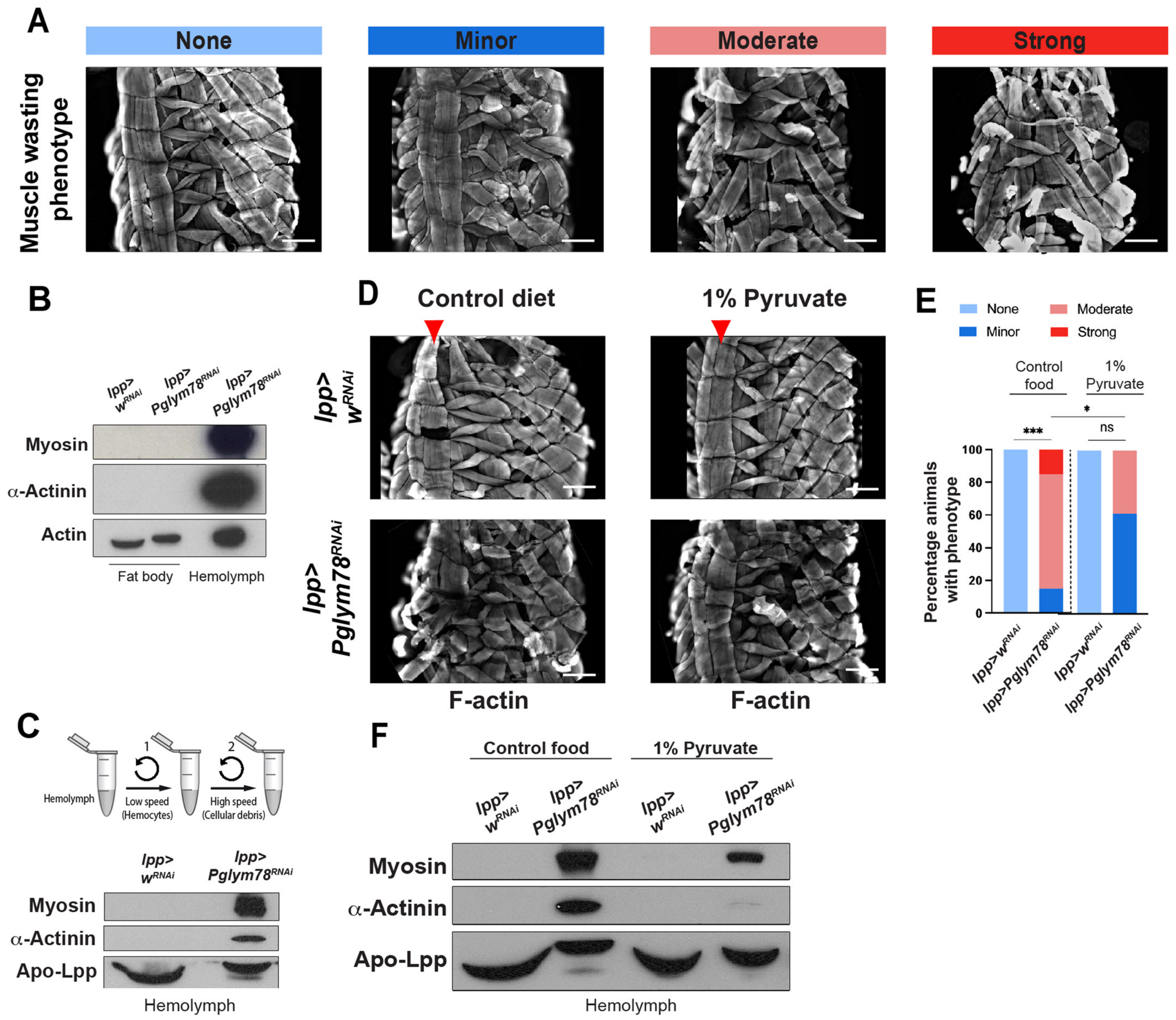

Figure EV3. Presence of α-actinin in the hemolymph of animals with fat body atrophy (related to Fig. 3).

(**A**) Categories used to quantify the muscle disorganization. White bar 200 μm. (**B**) Western blot of whole protein extracts of fat body tissues and hemolymph of the indicated genotypes monitoring the presence of muscular proteins Myosin and α-Actinin in the fat body. The hemolymph of *Ipp-Gal4> Pglym78-RNAi* animals is used as a positive control. Actin is used as a loading control between the two fat body samples. (**C**) Upper panel: experimental set-up to clear the hemolymph collected from bled larvae: a first centrifugation at low speed to remove circulating cells, and a second at high speed to remove cellular debris. Lower panel: western blot of whole protein extracts of hemolymph (5 μl samples) from *IppGal4> Pglym78-RNAi* larvae after centrifugations and monitoring the presence of muscle proteins Myosin and α-Actinin. Apo-LppII is used as a loading control. (**D**) Larval body wall muscles of *IppG4> Pglym78-RNAi* animals grown on normal food (left) or food supplemented with pyruvate (right) monitored using F-actin staining (white); white bar 200 μm. The red arrowhead indicates the VL3/VL4 muscles. In all panels anterior is up. (**E**) Quantification of the muscle defects from (**D**) and categorized according to severity. For the different genotypes, $n = 10$. Chi-square test, $***p = 0.0002$, $*p = 0.0156$, ns not significant. (**F**) Western blot of whole protein extracts of hemolymph (5 μl samples) from the larvae shown in (**D**) and monitoring the presence of muscle proteins such as Myosin and α-Actinin. Apo-LppII is used as a loading control. Source data are available online for this figure.

 © The Author(s)

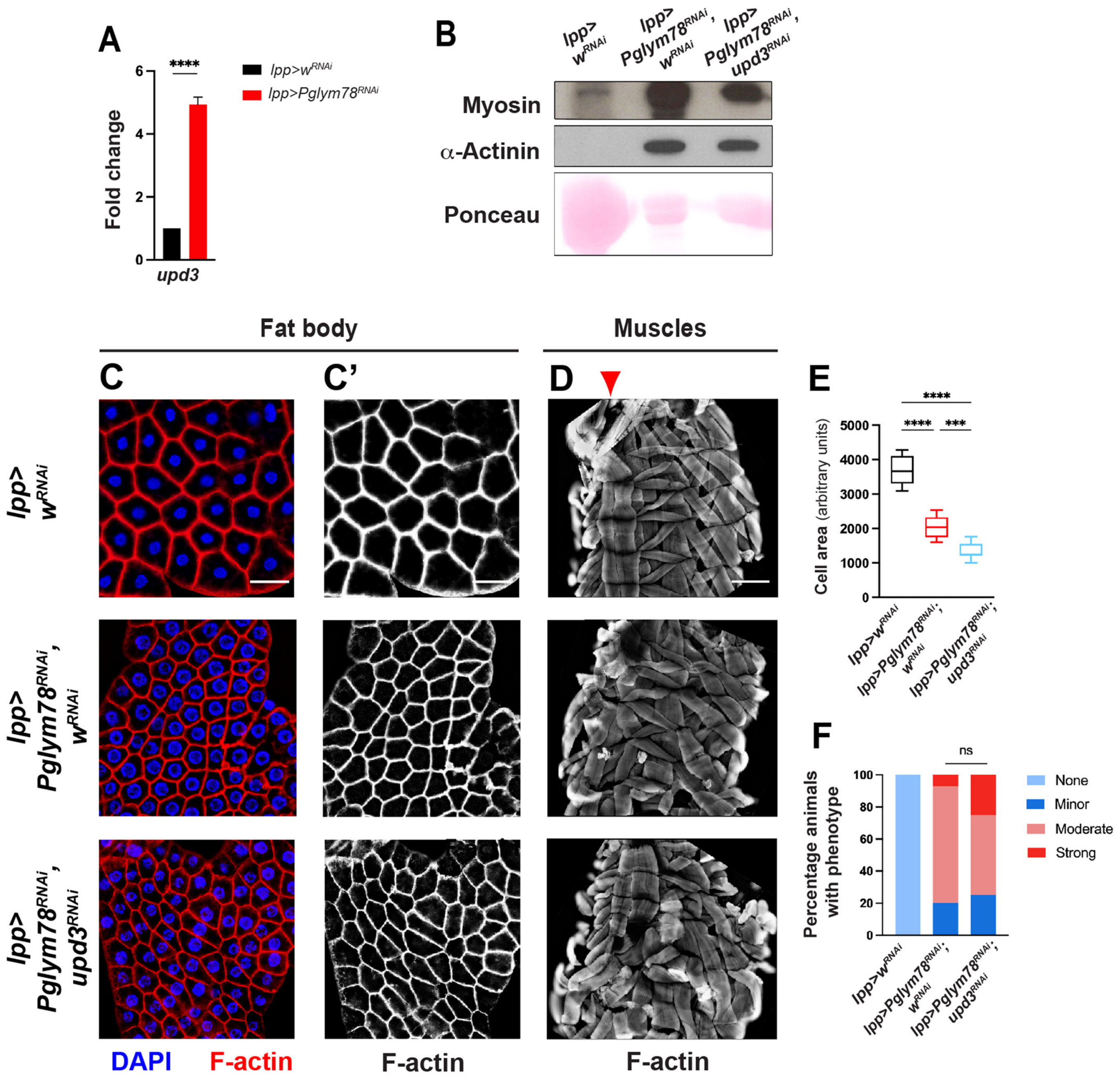

Figure EV4. Fat body-derived upd3 does not mediate the muscle disorganization triggered by fat body atrophy.

(**A**) Expression of the unpaired ligand *upd3* in fat body cells after *Pglym78* knock-down monitored by qRT-PCR. Reference gene for normalization: *rp49/RpL32*. Biological triplicates. Error bars show the standard error of the mean (sem). *T*-test, $****p < 0.0001$. (**B**) Western blot of whole protein extracts of hemolymph (5 μl samples) monitoring the presence of muscle proteins such as Myosin and α-Actinin. Ponceau S staining shows the Lsp proteins in the different samples and shows equivalent loading between animals with *Pglym78-RNAi* and animals with combined *Pglym78-RNAi & upd3-RNAi*. Of note, Lsp release is lower in animals with affected fat bodies. (**C**) Fat body staining of the indicated genotypes showing nuclei (DAPI, blue in **C**), cell cortex (F-actin, red in (**B**), and white in **C**'); white bar 50 μm. (**D**) Larval body wall muscles of the same genotypes as in (**C**) monitored using F-actin staining (white in **D**) on dissected fixed larvae; white bar 200 μm. The red arrowhead indicates the VL3/VL4 muscles. In all panels anterior is up. (**E**) Quantification of average cell size from images shown in (**C**), and represented has box plots where the whiskers represent the maxima and minima experimental points, the boxes represent the 25 and 75% percentiles, and where center line is the median. Biological replicates $n = 7$–11. One-way ANOVA statistical test, $****p < 0.0001$, $***p = 0.0003$. (**F**) Quantification of the muscle defects from (**D**) and categorized according to severity. For the different genotypes, $n > 10$. Chi-square test, ns not significant. Source data are available online for this figure.

© The Author(s)

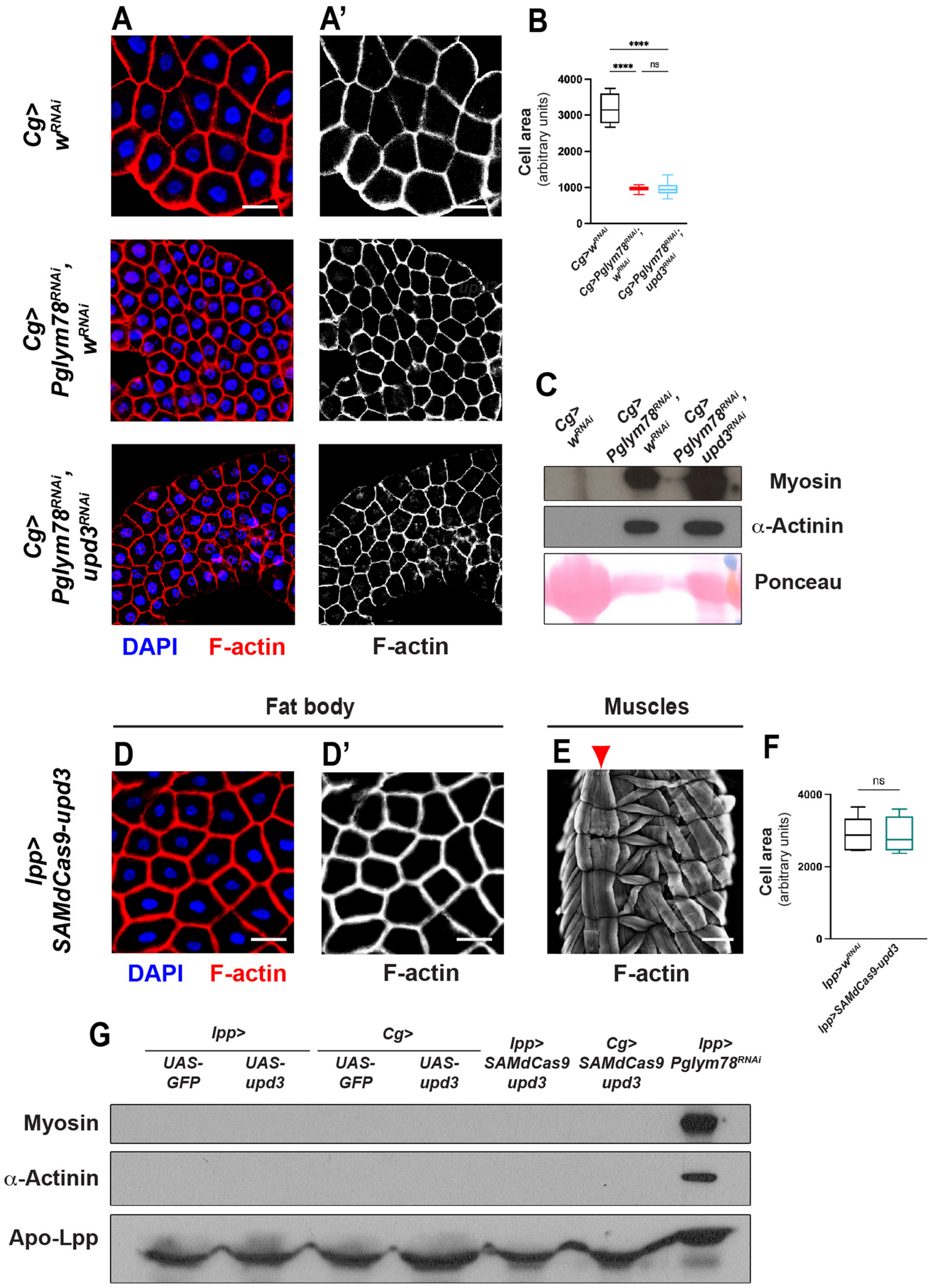

A
A'
B
C
Cg> w^RNAi
Cg> Pglym78^RNAi, w^RNAi
Cg> Pglym78^RNAi, upd3^RNAi
DAPI
F-actin
F-actin
Cell area (arbitrary units)
****
****
ns
Myosin
α-Actinin
Ponceau
Fat body
Muscles
D
D'
E
F
Ipp> SAMdCas9-upd3
DAPI
F-actin
F-actin
F-actin
ns
Ipp>w^RNAi
Ipp>SAMdCas9-upd3
G
Ipp>
Cg>
UAS-GFP
UAS-upd3
UAS-GFP
UAS-upd3
Ipp> SAMdCas9 upd3
Cg> SAMdCas9 upd3
Ipp> Pglym78^RNAi
Myosin
α-Actinin
Apo-Lpp

 © The Author(s)

Figure EV5. Fat body and hemocyte-derived Upd3 is neither required nor sufficient for muscle disorganization.

(**A**) Fat body staining after *Pglym78* invalidation in both adipocytes and hemocytes using the Cg-Gal4 driver, and showing nuclei (DAPI, blue in **A**) and cell cortex (F-actin, red in **A** and white in **A'**); white bar 50 µm. (**B**) Quantification of average cell size from images shown in (**A**), and represented has box plots where the whiskers represent the maxima and minima experimental points, the boxes represent the 25 and 75% percentiles, and where center line is the median. Biological replicates $n = 6–11$. One-way ANOVA statistical test, ****$p < 0.0001$, ns not significant. (**C**) Western blot of whole protein extracts of hemolymph (5 µl samples) from the larvae shown in (**A**) and monitoring the presence of Myosin and α-Actinin. Ponceau shows the Lsp proteins in the different samples. (**D**) Fat body staining after upd3 overexpression in adipocytes, and showing nuclei (DAPI, blue in (**D**)) and cell cortex (F-actin, red in (**D**) and white in **D'**); white bar 50 µm. (**E**) Larval body wall muscles of the same genotypes as in (**D**) monitored using F-actin staining (white in **E**) on dissected fixed larvae; white bar 200 µm. The red arrowhead indicates the VL3/VL4 muscles. Anterior is up. (**F**) Quantification of average cell size from images shown in (**D**), and represented has box plots where the whiskers represent the maxima and minima experimental points, the boxes represent the 25 and 75% percentiles, and where center line is the median. Biological replicates $n = 8$. Mann–Whitney test, ns not significant. (**G**) Western blot of whole protein extracts of hemolymph (5 µl samples) from 5d ael larvae overexpressing upd3 either in the adipocytes (*lpp-Gal4*), or in adipocytes and hemocytes (*Cg-Gal4*) and monitoring the presence of Myosin and α-Actinin. The right lane is hemolymph from *lpp-Gal4> Pglym78-RNAi* animals and is used as a positive control for the presence of Myosin and α-Actinin. Apo-LppII is used as a loading control. Source data are available online for this figure.
